# Characteristics of nursing care expanded by anthroposophy: a scoping review

**DOI:** 10.1590/1518-8345.7819.4792

**Published:** 2026-03-16

**Authors:** Susana Martín-Hernández, Janaina Meirelles Sousa, Georgina Casanova-Garrigos

**Affiliations:** 1 Universitat Rovira i Virgili, Campus Terres de l’Ebre, Facultat d’Infermeria, Tortosa, CT, Spain.; 2 Universidade de Brasília, Campus Ceilândia, Faculdade de Ciências de Tecnologias em Saúde, Ceilândia, DF, Brazil.

**Keywords:** Delivery of Health Care, Nursing Care, Nursing, Anthroposophy, Health Services, Complementary Therapies.

## Abstract

**Objective:**

to map the characteristics of Anthroposophy-enhanced nursing care in healthcare services.

**Method:**

a scoping review guided by the JBI. Eighteen sources of information were included, and original articles and gray literature published without restrictions concerning year or language were considered. After removing duplicates, the selection was conducted by two blind and independent reviewers. Data were extracted based on a script and presented in flowchart and table formats.

**Results:**

the 12 studies selected in this review showed that the characteristics of Anthroposophy-enhanced nursing care are related to the knowledge and skills for applying compresses, baths, foot baths, rhythmical *Einreibung*, pentagram body *Einreibung*, poultices, and medication administration in different healthcare settings, for acute or chronic conditions.

**Conclusion:**

Anthroposophy-enhanced nursing care in healthcare services is a promising specialty in the global scenario of integrative and complementary practices. In this sense, future studies are needed to validate it and to enhance the curative aspects of care. Register: https://doi.org/10.17605/OSF.IO/NV6D5

## Highlights

**(1)** Anthroposophic Nursing (AN) care can be integrated with conventional nursing. **(2)** Applicability of AN care to different clinical and health contexts. **(3)** The need for knowledge and skills derived from Anthroposophic Medicine. **(4)** The appreciation of the curative aspects of care implicit in nurses’ approach. **(5)** The potential for action in the global health scenario and WHO reference criteria.

## Introduction

Anthroposophic Nursing (AN) care is part of the Anthroposophic Medicine (AM) medical system and is based on conventional nursing approaches and competencies, expanded by anthroposophical knowledge of the human being. The anthroposophical differentiation between the physical body, vital forces, soul, and spirit, and the understanding of biographical development throughout the life course provide additional perspectives on nursing competencies, processes, and attitudes^1^ ^-^ ^2^.

AM is an integrative medical system that extends conventional medicine with cognitive methods and anthroposophy concepts established by Rudolf Steiner and physician Ita Wegman in 1920. In this system, life interactions expressed by the body, soul, and spirit are regulated according to each person’s biography, social, and environmental context, and can be supported by treatments and therapies through medication, nursing care, psychotherapy, art therapy, therapeutic eurythmy, and movement and body therapies. The treatments and therapies offered by MA are targeted at the individual, family, community, and caregivers, in line with the health needs presented in the context of health services. This system highlights the possibility of making the spiritual understanding of the human being verifiable and documentable, through the assessment and articulation of aspects not only of the body, but also of the soul and spirit^3^ ^-^ ^4^.

According to anthroposophy, the human being has a fourfold nature: the physical, etheric, or vital body, the astral, or soul body, and the spiritual, or “I.” The physical body is everything that can be touched and measured, such as vital signs, laboratory results, height, and weight. The vital body is active in the body’s regenerative processes and rhythms, such as digestion and sleep. The astral body (soul) has a strong relationship with thoughts and emotions. Finally, the human “I” manifests itself in biography, life paths, destiny, and spiritual pursuits as a conscious aspect of the Self^4^. AM is practiced in primary, secondary, and tertiary care settings, rehabilitation centers, nursing homes, community nursing services, anthroposophic hospitals and clinics, public hospitals, and university medical centers. The approach is applied in an integrated manner with conventional Western medicine and in conjunction with other medical specialties^1^ ^-^ ^2^.

The Anthroposophic nursing team, in partnership with those providing AM care, has the highest level of integrative medicine penetration in Germany and Switzerland, offering therapies in anthroposophic hospitals and academic university hospitals. Their practice is recognized by the German and Dutch Nursing Councils^1^ ^,^ ^3^. 

In Brazil, Anthroposophic Medicine is part of the list of Integrative and Complementary Practices institutionalized in the Unified Health System (SUS) through the National Policy for Integrative and Complementary Practices (PNPIC). Anthroposophy applied to health was recently recognized by the Federal Nursing Council as an area of nursing competence and practice^5^ ^-^ ^6^. Recently, AN became part of the World Health Organization (WHO) Benchmarks for Anthroposophic Medicine, which outline standards and criteria for the training and practice of healthcare professionals^1^.

As countries establish regulatory frameworks for the practice of Integrative and Complementary Therapies, policymakers need information for decision-making, including assessment of the quality of practices, challenges, and implementation approaches^1^.

Therefore, after a preliminary search of MEDLINE (via PubMed), JBI Evidence Syntheses, and the Cochrane Database of Systematic Review, no published or ongoing systematic or scoping reviews were identified that investigated the characteristics of anthroposophy-enhanced nursing care and its practices. That being so, this study aims to map the characteristics of anthroposophy-enhanced nursing care in health services^7^ ^-^ ^9^.

## Method

### Study design

This is a scoping review guided by the JBI and the PRISMA Extension for Scoping Reviews (PRISMA-ScR): Checklist and Explanation^7^ ^-^ ^9^. To record development intention and scientific availability, the protocol was registered on the Open Science Framework platform and can be accessed through DOI 10.17605/OSF.IO/NV6D5^8^. This review aimed to answer the guiding question: “What evidence is there in the literature on the characteristics of nursing care expanded by Anthroposophy in health services?” This question was developed using the acronym PCC, with the population (P) being people at any stage of the human life cycle, with regard to the concept (C) and characteristics of nursing care expanded by Anthroposophy, and with regard to the context (C), health care settings.

### Period

Data searches took place between March and April 2024.

### Selection criteria

The inclusion criteria were: original articles and gray literature published without restriction on year of publication or language. Articles not available in full, whose titles and abstracts did not answer the guiding question, as well as letters to the editor, editorials, review articles, books/book chapters, and conference abstracts were excluded.

### Search strategy

Regarding the research strategy and identification of studies, the following electronic information sources were used: Academic Archive Online (DiVA), Academic Search Premier (EBSCOhost), CINAHL with full text (via EBSCOhost), *Der Merkurstab*, EMBASE (Elsevier), LILACS (BVS), MEDLINE (via PubMed), MOSAICO (*Modelos de Saúde e Medicina Tradicionais, Integrativas e Complementares nas Américas* - via BVS), SciELO (Scientific Electronic Library Online), Scopus (Elsevier), ScienceDirect (Elsevier), Web of Science - Core Collection (Clarivate Analytics). The journal *Der Merkurstab* was accessed via the Anthromedics website, a virtual environment of reference for publications in the field of Anthroposophic Medicine. The search for gray literature (dissertations and theses) was conducted on the platforms of the Brazilian Digital Library of Theses and Dissertations (BDTD), the Catalog of Theses and Dissertations of the Coordination for the Improvement of Higher Education Personnel (CAPES), CyberTesis, NDLTD (Global ETD Search), the Open Access Scientific Repository of Portugal (RCAAP), and Theses Canada.

The search strategy was developed with the support of a librarian, using the Boolean operators AND and OR in Portuguese, English, and Spanish. Various combinations of descriptors extracted from the Medical Subject Headings (MeSH) and Health Sciences Descriptors (DeCS) were tested. These descriptors were combined into a search strategy adapted to the specificities of each information source/repository consulted in the review, as illustrated in Figure 1. The survey of publications in the aforementioned information sources took place on April 9, 2024.

**Figure 1:** Search strategies developed, adapted to each data source. Brasília, DF, Brazil, 2024

**Data source**	**Search strategy**
Academic Archive Online (DiVA)	Anthroposop* AND Nursing
Academic Search Premier (EBSCOhost)	(SU “Anthroposop*”) AND (SU “Nursing Care” OR “Nursing”) AND Health Care
*Biblioteca Digital Brasileira de Teses e Dissertações* **(BDTD)**	‘Anthroposop* AND (“Nursing Care” OR “Nursing”)’
*Catálogo de Teses e Dissertações CAPES*	Antroposofia e Enfermagem
*CyberTesis*	“Anthroposophical” OR “Anthroposophic” OR “Anthroposophy” OR “Antroposofia”
CINAHL with full text (via EBSCOhost)	(TI “Anthroposop*”) AND (“Nursing Care” OR “Nursing”) AND Health Care
*Der Merkurstab* **(via Anthromedics)**	Nursing
EMBASE (Elsevier)	‘anthroposop*’ AND (‘nursing care’/exp OR ‘nursing care’ OR ‘nursing’/exp OR ‘nursing’) AND (‘health care’/exp OR ‘health care’ OR ((‘health’/exp OR health) AND (‘care’/exp OR care)))
LILACS (BVS)	(db:”LILACS”) AND (Anthroposop*) AND (Nursing)
MEDLINE (via PubMed)	(“Antroposofia”[Título/Resumo] OR “Antroposop*”[Título/Resumo]) AND (“Cuidados de Enfermagem”[Título/Resumo] OR “Atencion de Enfermeria”[Título/Resumo] OR “Cuidados de Saúde”[Título/ Resumo] OU “Enfermagem”[Título/Resumo])
MOSAICO (*Modelos de Saúde e Medicinas Tradicionais, Complementares e Integrativas nas Américas* - via BVS)	(anthroposop*) AND (nursing) AND ( collection_mtc:(“MTYCI”))
NDLTD (*Global ETD Search*)	“Anthroposophical” OR “Anthroposophic” OR “Anthroposophy” AND Nursing
Repositório Científico de Acesso Aberto de Portugal (RCAAP)	Anthroposophical OR Anthroposophic OR Anthroposophy AND Nursing
SciELO	(Anthroposophical OR Anthroposophic OR Anthroposophy ) AND (Nursing)
Scopus (Elsevier)	“Anthroposop*” AND ( “Nursing Care” OR “Nursing” ) AND health AND care
ScienceDirect (Elsevier)	(“Anthroposophical” OR “Anthroposophic” OR “Anthroposophy”) AND (“Nursing Care” OR “Nursing”)
Theses Canada	Anthroposop* AND Nursing
Web of Science - Core Collection (Clarivate Analytics)	“Anthroposop*” (Topic) AND (“Nursing Care” OR “Nursing”) (All Fields) AND Health Care (All Fields)

### Study selection

After the search, the studies were forwarded to the EndNoteWeb manager for archiving and exclusion of duplicate articles. Second, the references were sent to the Rayyan^®^-Intelligent Systematic Review software^10^, accessed by two reviewers to read titles and abstracts, and then the studies were classified using the inclusion and exclusion criteria. In case of disagreement, a third reviewer was consulted. In the next step, the selected studies were read in full and further screened using the inclusion and exclusion criteria. 

### Data extraction and presentation

Data extraction was performed using an extraction form based on the JBI recommendations^11^, adapted by the authors with the information of interest for this review, namely: a) Bibliographic information and study characteristics: title, author(s), year, study location (country), general objective, type of study; b) Characteristics of nursing care augmented by anthroposophy: characteristics of nursing interventions performed in care settings (types, duration, frequency), health service where nursing interventions are implemented, outcomes, and recommendations.

The synthesis of the results was illustrated in the form of a flowchart and tables, followed by a narrative summary of the mapped results, describing how they relate to the research objective and question. The study and methodological quality were not assessed, as the purpose of this scoping review was not to provide a critical analysis of the evidence. 

## Results

**Figure 2:**
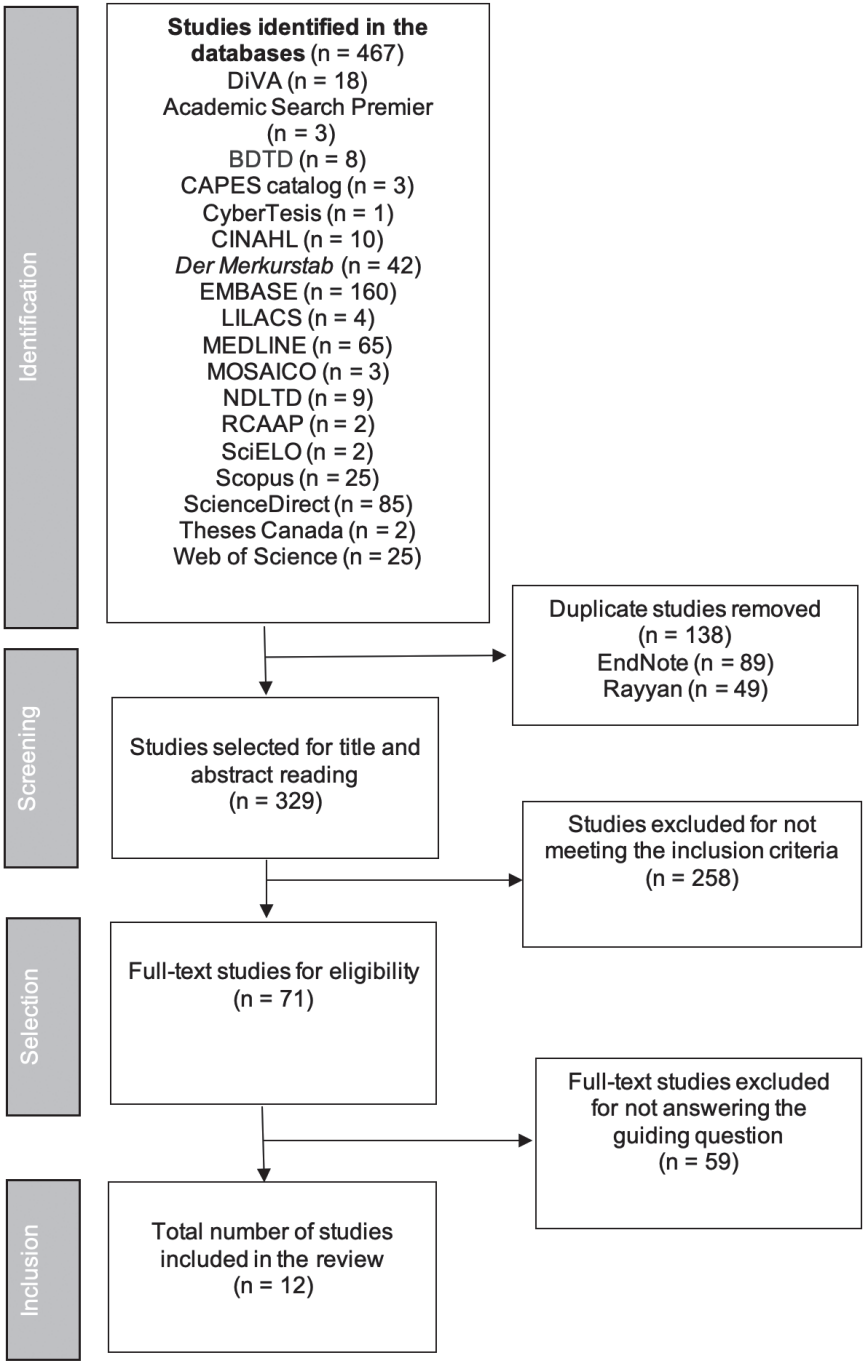
Study selection flowchart according to PRISMA recommendations. Brasília, DF, Brazil, 2024

After paired analysis and double-blind selection by reviewers, 12 publications were included for review as shown in Figure 2.

Figure 3 presents the studies selected for scoping review, according to bibliographic information and study characteristics.

**Figure 3:** Characterization of mapped publications according to authors, article title, country and year, type of study, sample, objective, and health service. Brasília, DF, Brazil, 2024

Study code	Authors, translated title of the article, country and year	Type of study and sample	Objective	Health service
E1^12^	Bräuner G, Gerber W, Schulmayer H. Inpatient treatment of a 7-year-old girl with chronic constipation and epilepsy. A case report from a therapeutic eurythmy, nursing, and medical perspective. Germany 2004.	Case study: 7-year-old girl with chronic constipation and seizures.	To describe the treatment of a 7-year-old girl with chronic constipation and epilepsy from the perspective of therapeutic eurythmy, nursing, and medicine.	Anthroposophical Hospital
E2^13^	Dach C, Heine R, Heiligtag HR. Anthroposophical care for cancer patients, Germany 2009.	Case study. Female patient diagnosed with invasive ductal breast cancer on the left side, underwent conservative mastectomy and lymphadenectomy.	Describe nursing therapies in the treatment of cancer patients at all stages of the disease.	Anthroposophical Clinic
E3^14^	Stüdemann G. Borago compresses for lymphedema. Observations from a nursing perspective, Germany 2011.	Qualitative research using a questionnaire. Seventeen cancer patients with lymphedema.	To identify the perception of well-being of patients with lymphedema, after treatment with *Borago officinalis* compress.	Anthroposophical Hospital
E4^15^	Ribeiro RM. Care expanded by anthroposophy: a case study on the practice of Anthroposophical nursing, Brazil 2013.	Case study with participant observation. Nursing team (1 nurse and 1 nursing technician).	To describe the practice of Anthroposophic Nursing based on its practice and work experience in an anthroposophic medical clinic; to identify the treatments adopted in Anthroposophic Nursing care; to analyze the foundations of care provided by nursing to human beings.	Anthroposophical Clinic
E5^16^	Therkleson T. Topical ginger treatment with a compress or patch for osteoarthritis symptoms, New Zealand 2014.	Randomized study. Twenty adults with chronic osteoarthritis.	To assess and compare participants’ perceptions of pain intensity, chronic fatigue, overall effect of osteoarthritis, mobility, and health satisfaction; use of pain medication and/or pain management; safety of the application of the patch and ginger compress.	Anthroposophical Clinic and Home
E6^17^	Therkleson T. Ginger therapy for osteoarthritis: a typical case, New Zealand 2014.	Case study. Patient with chronic osteoarthritis.	To describe treatment for osteoarthritis with ginger therapy, applied by anthroposophic nurses for a specific personality type.	Anthroposophical Clinic
E7^18^	Therkleson T. A 12 year old’s search for self: case report, New Zealand 2014.	Case study: 12-year-old boy with complex post-traumatic stress disorder (C-PTSD).	To describe the effects of anthroposophic nursing treatment on a boy with complex post-traumatic stress disorder.	Anthroposophical Clinic
E8^19^	Kusserow M. External applications in anthroposophic practices and clinics, Germany 2014.	Case study: 5-year-old boy with bilateral pneumonia.	Describe the anthroposophic treatment for a 5-year-old boy with bilateral viral pneumonia.	Homeopathy Unit of a Regional Hospital
E9^20^	Therkleson T, Stronach S. Broken Heart Syndrome: A Typical Case, New Zealand 2015.	Case study. An 82-year-old woman with “Broken Heart Syndrome,” general exhaustion, and anxiety attacks.	Describe treatment for Takotsubo cardiomyopathy “Broken Heart Syndrome” “.	Medical Center and Home
E10^21^	Ström M. Rhythmical *Einreibung* according to Wegman/Hauschka - an interview study on patients’ experiences of touch therapy in outpatient care, Sweden 2016.	Content analysis. Five patients over 18 years of age.	To clarify how patients who received rhythmical *Einreibung* in outpatient care experienced the treatment, and how it affected them.	Anthroposophical Outpatient Clinic
E11^22^	Deckers B, Schoen-Angerer T, Voggenreiter B, Vagedes J. External nursing applications in the supportive management of prolonged postoperative ileus: description of interventions and case report, Germany 2016.	Case report. A 61-year-old male patient with prolonged postoperative ileus.	To describe the anthroposophical approach to nursing for the treatment of prolonged postoperative ileus.	Anthroposophical Hospital
E12^23^	Fujiwara-Pichler E, Beckmann U, Madeleyn R. Community-acquired bacterial pneumonia in a child treated without antibiotics in a hospital for anthroposophic medicine: a case report, Germany 2020.	Case study. A 4.5-year-old Caucasian boy presented with community-acquired bacterial pneumonia (CAP) preceded by a mixed viral and streptococcal throat infection.	To describe the treatment of community-acquired bacterial pneumonia in a child treated without antibiotics in an anthroposophic medicine hospital.	Anthroposophical Hospital

Nursing care expanded by anthroposophy is characterized in Figure 4, through the visualization of the type, duration and frequency, results and recommendations described in the studies. 

**Figure 4:** Characterization of nursing interventions according to type, duration, frequency, results, and recommendations. Brasília, DF, Brazil, 2024

Study code	Clinical data	Type, duration and frequency of nursing care	Results and recommendations
E1^12^	7-year-old girl with chronic constipation and seizures.	Abdominal massage with 10% Oxalis folium ointment in a circle around the navel for about 5 minutes; 10% lavender oil dispersion bath with a 10-minute rest after the bath; 10% lavender oil dispersion on the legs and feet with a 10-minute rest. The dispersion baths were performed on alternate days; the abdominal massage was applied every day before lunch; and the legs and feet massage was applied every day during the hospitalization period.	Nursing care combined with art therapy, rhythmic massage, elements of curative education, family-oriented conversation, and medical treatment allowed the constipation to be completely cured after three weeks of hospitalization, with improvement in seizures and harmonization of the family situation.
E2^13^	Woman with breast cancer undergoing conservative mastectomy and lymphadenectomy.	During the first week of hospitalization, the following were performed: rhythmic back massage with *Solum ulioginosum* oil; liver compress with *Achillea millefolium* tea after lunch; left arm wrap with Aconitum oil; and arnica compress on both breasts to relieve tension, pain, and heaviness. During the second week of hospitalization, the following were performed: rhythmic back massage and Quark cheese wrap on the left breast and chest. The anthroposophic nursing gestures identified in the first two weeks were relieving, embracing, and comforting; in the following weeks, they were creating space and relieving.	The patient experienced improvement in pain in her arm, breast, and back, as well as the sensation of tension and heaviness in her left arm and breast, without the need for analgesics or sleeping pills upon discharge. She was taught how to apply compresses and wraps at home for neck pain. External anthroposophic therapies are recommended as a complement and extension of oncological care.
E3^14^	Cancer patient with lymphedema.	Application of a compress with 20% *Borago officinalis L*. Essence for 30 minutes on the affected limb, and then application of *Angelica archangelica* ointment to the congested extremities, once a day, for 2 to 3 weeks or until the edema reduces.	The application of compresses and ointments brought significant relief from the symptoms arising from lymphedema, as well as the body’s perception of being “lighter”, “relaxed”, “thinner legs”, and “refreshed”.
E4^15^	Nursing practice in an Anthroposophical clinic.	Intestinal baths with herbal tea, baking soda, coarse salt, silica, and Formica; oil dispersion baths; organ compresses with solutions and teas; foot baths with teas, tinctures, and salts; body rhythmical *Einreibung*; wrapping the entire body with a heated sheet; and parenteral medication administration. The duration of the therapies varied depending on the treatment applied, averaging thirty to sixty minutes. After the therapy, the patient remained in a quiet, restful environment for the regenerative phase to occur. The frequency of interventions varied, guided by the physician’s prescription.	The therapies identified were intestinal baths, compresses, body rhythmical *Einreibung*, oil immersion baths, wraps, foot baths, and the administration of *Viscum album* therapy to cancer patients. The care was based on the twelve senses, the seven vital processes, the twelve gestures of care, and the three-membered and four-membered. It emphasizes the patient as the focus of care, and that the healing process involves the existence of subtle forces that can be stimulated by nursing care.
E5^16^	Adult patients with chronic osteoarthritis.	Application of a patch made from ground dried organic matter of *Zingiber officinale* rhizome or a ginger compress to the mid-lumbar region for 30 minutes, followed by a 20-minute rest. Topical application of a ginger patch or compress once daily for 7 consecutive days; topical self-application of a ginger patch at home for 24 weeks.	After 1 week of topical ginger treatment, there was a decline in scores for pain, fatigue, global effect, and functional status, while health satisfaction improved from 80% dissatisfied to 70% satisfied. Scores in all five domains progressively decreased over the course of 24 weeks of self-treatment, as did the reduction in analgesic use, with most participants no longer using pain medication at the end of the study period. Topical ginger treatment is recommended because it is simple and cost-effective to apply and should be considered in the care of elderly patients with chronic osteoarthritis.
E6^17^	Patient with osteoarthritis.	Application of a ginger compress to the mid-lumbar (renal) region for 30 minutes, followed by a 15-minute rest, for 7 consecutive days, and continuous applications two or three times a week, to maintain pain levels.	The ginger compress provided immediate and progressive relief of osteoarthritis symptoms over 24 weeks, with no negative effects reported by the patient. The use of ginger compresses is recommended in nursing care for patients with osteoarthritis.
E7^18^	12-year-old boy with complex post-traumatic stress disorder.	Combined therapy of lemon footbath, rhythmical *Einreibung* with *Solum ulioginosum* oil, cardiac massage with Aurum-Lavender-Rose ointment, and pentagram *Einreibung* with Aurum-Lavender-Rose ointment. The therapies were applied once a week for 5 weeks in 60-minute sessions, with 15 minutes of rest between treatments.	Foot baths, cardiac massage, and rhythmical *Einreibung*, indicated for the symptoms of complex post-traumatic stress disorder, only had an effect after the inclusion of pentagram *Einreibung*. It is recommended that pentagram *Einreibung* be considered in the management of trauma, stress, exhaustion, and disconnection in 12-year-old children.
E8^19^	5-year-old boy with bilateral pneumonia.	Application of *Cochlearia armoracia* gel to the sinuses; *Achillea millefolium* compress with *Petacites officinalis* on the chest area for 45 minutes, once a day, for 14 days. Lemon compress during fever.	The compresses brought relief from the symptoms of coughing and difficulty breathing, relaxation for sleeping, recovery of appetite, improvement in mood - becoming happier, and self-regulation of temperature.
E9^20^	82-year-old woman with “Broken Heart Syndrome”.	Lavender foot baths, rhythmical *Einreibung* using *Solum ulioginosum* oil, and a warm poultice of *Oxalis folium* ointment were performed once a week for four weeks. At home, during treatment, the patient received a “heart pillow” impregnated with Aurum Lavender-Rose ointment, to be applied to the left breast at night, for daily use for four weeks.	The foot bath and rhythmical *Einreibung* treatment did not interfere with the response to the medications being used, and style="padding:10px; border:1px solid black" allowed the traumatized elderly woman to overcome her fear and find renewed courage to respond to her world and that of others. Foot baths and rhythmical *Einreibung* are recommended when there are limited options for managing trauma and stress.
E10^21^	Patients who received rhythmical *Einreibung*.	Rhythmical *Einreibung* sessions in an outpatient setting, applied once or twice a week, for 4 to 8 weeks.	Rhythmical *Einreibung* led to improvements in lung function (cough, asthma, and chronic obstructive pulmonary disease), bowel function, headache, general pain in prolonged pain, and sleep. Experiencing the care provided was described as feeling stability, security, a sense of connection, and relaxation. The experience of being affirmed was described as recovering boundaries, being seen, feeling oneself, feeling meaningful, and participating in a context. Vitality was expressed as experiences of feeling stronger, hopeful, and more energetic. Experiences of health despite suffering and serious illness were described as feeling good and calm in a chaotic life situation.
E11^22^	Patient with prolonged postoperative ileus.	Application of a compress with 20% *Oxalis folium* tincture to the abdominal region for 20 minutes, followed by 30 minutes of rest; massage with lemon balm oil on the abdomen, in a clockwise direction, for approximately 5 minutes; application of a compress with *Thuja occidentalis* ointment 10% and *Argentum Metallicum* 0.4%, in equal amounts, to the abdomen for 30 minutes, followed by 30 minutes of rest. For 10 days, an *Oxalis folium* compress was applied once in the morning followed by a lemon balm oil massage; and once at night, a *Thuja*/*Argentum* ointment compress was applied.	The use of compresses and abdominal massage improved the sense of well-being and reduced pain, making it possible to manage prolonged postoperative ileus without prokinetic drugs. Compresses and light abdominal massage are recommended as additional tools in the management of prolonged postoperative ileus.
E12^23^	4.5-year-old boy with community-acquired bacterial pneumonia.	Application of a warm Quark cheese compress to the right chest wall for 20 minutes on the first day of treatment; application of a warm lavender oil compress to the anterior chest wall once a day for 6 days; application of a hot water bottle for hypothermia after rectal medication for fever; from the second to the sixth day of treatment, a ground mustard compress was applied to the right chest wall for 2 to 3 minutes.	Pneumonia was treated effectively and safely without antibiotics, with pediatricians trained to reevaluate the child frequently, nursing staff familiar with this treatment, and parental support. It is recommended that therapies be administered in appropriate and specialized facilities for this type of treatment, including highly trained personnel to reduce the risk of complications, including death.

## Discussion

Among the studies analyzed in this review, regarding language, six were written in English, four in German, one in Portuguese and one in Swedish. Regarding the type of publication, ten correspond to scientific articles, one a master’s dissertation and one a doctoral thesis. Regarding geographic origin, four studies came from New Zealand, six from Germany, one from Brazil and one from Sweden^12^ ^-^ ^23^. 

This review made it possible to map the characteristics of care expanded by anthroposophy, showing that EA uses external therapies, expressed in the practices of compresses, poultices, wraps, baths, foot baths, intestinal washes, honey massage, rhythmical *Einreibung*, pentagram *Einreibung*, parenteral and inhalation administration of medications, used for physical complaints (pain, cough, asthma, constipation, difficulty breathing, convulsions, muscle tension, fatigue, lack of appetite, difficulty in self-regulating temperature, pneumonia) and emotional complaints (fear, psychological suffering, dissatisfaction with health, stress, mental exhaustion, depressed mood). These therapies combine interpersonal attention, touch, rhythmic movements, heat maintenance, and herbal elements (oil, essences, tinctures, ointments) in interventions applied daily or once or twice a week, in 30-60-minute sessions, the duration depending on the substance applied, followed by a period of therapeutic rest. They are administered in treatment cycles according to the patient’s clinical condition^12^ ^-^ ^23^.

In the studies analyzed, external therapies were combined with anthroposophic drug therapy at the beginning of treatment, aiming to stimulate the self-regulation of body heat and the mobilization of etheric forces. These act as a vehicle for medication access to other bodies, the soul and the self, contributing to the faster effect of oral medication and the mobilization of forces that aid in the healing process^12^ ^,^ ^14^ ^,^ ^16^ ^,^ ^18^ ^-^ ^20^ ^,^ ^22^ ^-^ ^23^. Initially, external therapies are adjuvants in the therapeutic process, but as sessions progress, they become the main curative therapy^13^ ^,^ ^16^ ^-^ ^17^. External therapies are highlighted in the literature as facilitators of autonomic self-regulation and salutogenic processes (which promote and enhance the development of a good subjective state of health), physically influencing the distribution of heat in the organism, integrating the functions of body, soul, and spirit through the regulation of vital processes such as respiration, heating, nutrition, excretion, maintenance, growth, and reproduction, as well as the harmonization of emotional issues and processes of meaning in life and health^2^ ^,^ ^24^.

Studies point to the individual as the focus of care in AN, through recognition of their fourfold nature^12^ ^-^ ^23^. The literature explains that by observing how the aspects of this fourfold nature interact with each other and how they are expressed, a new level of understanding is achieved, and a process of empirical evaluation of the more subtle bodies occurs. For example, the functionality of the vital body, which maintains all parts in a living relationship, can be seen in fluid balance, sleep/wake cycles, and the quality of hair and nails. The health of the emotional body can be assessed by emotional balance, experiences of pain, and strong reactions to circumstances or people with sympathy or antipathy. Finally, the presence of the “self” as active in the individual can be observed through the sense of coherence in life, the connection with spirituality and/or the world, the ability to become an observer of both others and oneself, and the ability to find meaning in life or illness. This provides the anthroposophical nurse with a deep understanding of others and themselves, and naturally informs the care provided^4^ ^,^ ^24^.

Studies have highlighted that AN care is offered to patients across different life cycles, with diverse clinical conditions, both acute and chronic, with interventions that produce immediate and short-term health impacts, in primary care, outpatient, and hospital settings, and that can be integrated with conventional medical practices^12^ ^-^ ^23^. Shared decision-making and an emphasis on self-management of disease-related symptoms were also highlighted, with medium- and long-term results. This is made possible by the possibility of some external therapies being taught for self-administration at home^13^ ^,^ ^16^ ^-^ ^17^ ^,^ ^20^, especially for patients experiencing chronic symptoms, due to their simple implementation and the low cost of their therapeutic elements.

In other settings, care has been evidenced, such as for chronic illnesses in children. Chronic illnesses are considered to stem from current lifestyles and sensory and mental experiences in childhood, and can be treated with anthroposophic therapy, especially when other therapies have been unsuccessfully attempted. The goal is to replace influences that cause illness with others that preserve health. The mutual integration of nursing therapies, art therapy, rhythmical *Einreibung*, curative education, guided discussions with the family, and pharmacological therapy contribute to creating a balance in the empathic perception of the individual family burden and a clear identification of the conditions and consequences of the influences of daily family life on children’s health. In this context, it is recognized that sometimes outpatient treatment does not produce the desired effect, requiring follow-up in a hospital setting so that physical and emotional aspects can be understood and monitored with a more assertive therapeutic plan for the child and the family context^12^. 

AN care in oncology has been highlighted through external therapies such as compresses, wraps, rhythmical *Einreibung* according to Wegman and Hauschka, anthroposophic medications, home health care guidelines, and the nurse’s internal attitude, which complement oncological care. Therapy in the oncological context was based on the promotion of internal warmth, establishing integration between the four human bodies and stimulating the healing principle. It is noteworthy that the external therapies offered arise from the nurse’s decision, based on the twelve gestures of anthroposophic nursing, which offer a model for assessment, guidance, and execution of the nursing therapeutic plan^13^ ^-^ ^14^.

The curative aspects of AN care and patient involvement are seen in the literature as intangible activities that contribute to positive patient outcomes and experiences, and need to be valued in healthcare settings with equal importance to conventional therapeutic healing processes^4^.

Studies show that the level of awareness nurses share in their interventions elevates nursing work beyond mechanical models to a therapeutic level, encompassing the intangible inner qualities nurses bring to the care experience. This expands awareness not of what the nurse does for the patient, but of how it is done. This process enables nurses to understand how the care provided affects the patient’s four bodies, in addition to influencing their internal mood and the professional’s own state of being. Each encounter is seen as unique, carrying within it the potential for transformation for all involved in the act of care. Conscious intention can be exemplified by attitudes expressed in 12 nursing gestures, which strengthen the nurse and support the patient^13^ ^,^ ^15^ ^,^ ^19^ ^,^ ^21^. The twelve Anthroposophic Nursing gestures are classified into two groups: engaging gestures and activating gestures. Engaging gestures aim to satisfy a need that a person, under a given condition, is unable to meet independently. In this approach, the nurse acts interventively, in the name of patient safety or comfort. Activating gestures, on the other hand, aim to encourage patients to become aware and act on their own behalf, fostering the recovery of their internal capacity for reaction and self-regulation^4^.

Given the findings, the limitation of this study is its scope, exploring publications that described nursing care expanded by anthroposophy, which limited the expressiveness of the review, as some studies focused on external therapies, but with unclear methodological designs that did not allow identifying whether the referred therapy was used in nursing practice. It is recommended that future studies focus on an objective and clear methodological approach and expand the scope of nursing practice to include management, teaching, and healthcare specialties referenced in the literature but not previously covered, such as Anthroposophic Obstetric Nursing^1^.

The results of this review contribute to the advancement of scientific knowledge in the field of Integrative and Complementary Practices, especially anthroposophic nursing. This field requires the enhancement of specialized skills and knowledge so that professionals can incorporate new technologies and practices, as well as essential interpersonal skills to deal with the diverse situations presented by the profession. The evidence collected helps fill the knowledge gap regarding the practice of Anthroposophic Nursing and highlights the need for future research to consolidate this knowledge and expand nursing care approaches in different healthcare contexts. 

## Conclusion

The mapping of the characteristics of nursing care expanded by Anthroposophy in health services highlighted Anthroposophic Nursing as a specialty within the nursing field, with a practice based on its own knowledge derived from Anthroposophic Medicine, which enables integration with conventional medical practices and multidisciplinary teamwork. It presents the individual as the focus of care, in different health settings, whether hospital, health clinic, or home, with diverse clinical conditions, both acute and chronic.

Nursing care requires nurses to have specific knowledge and skills regarding anthroposophic therapeutic practices to provide effective and safe care in different health and life contexts. These practices are designed for short-, medium-, and long-term follow-up, and some can be taught to patients for home use as a complement to the established therapeutic plan. The emphasis is on co-responsibility in the therapeutic process and the nurse’s attitude, perceived by patients as a facilitator of the healing process. Anthroposophic Nursing broadens the perspective on health promotion and individual needs of patients, which are sometimes beyond the limits of conventional medicine.

## Data Availability

All data generated or analysed during this study are included in this published article.
