# Supplementary material for: Characteristics of nursing care expanded by anthroposophy: a scoping review
Source: Rev Lat Am Enfermagem. 2026 Mar 16;34:e4792. doi: 10.1590/1518-8345.7819.4792 (PMC12991389; doi:10.1590/1518-8345.7819.4792)
Supplement: Translation

# Translation: Características do cuidado de enfermagem ampliado pela antroposofia: revisão de escopo

**DOI:** 10.1590/1518-8345.7819.4793

**Authors:** Susana Martín-Hernández, Janaina Meirelles Sousa, Georgina Casanova-Garrigos

**Affiliations:** 1 Universitat Rovira i Virgili, Campus Terres de l’Ebre, Facultat d’Infermeria, Tortosa, CT, Espanha.; 2 Universidade de Brasília, Campus Ceilândia, Faculdade de Ciências de Tecnologias em Saúde, Ceilândia, DF, Brasil.

**Keywords:** Atenção à Saúde, Cuidados de Enfermagem, Enfermagem, Medicina Antroposófica, Serviços de Saúde, Terapias Complementares.

## Abstract

**Objetivo:**

mapear as características do cuidado de enfermagem ampliado pela Antroposofia nos serviços de saúde.

**Método:**

revisão de escopo orientada pelo JBI. Foram incluídas 18 fontes de informação e considerados elegíveis artigos originais e literatura cinzenta, publicados sem restrição ao ano de publicação ou idioma. Após remoção de duplicatas, a seleção foi conduzida por duas revisoras de forma cega e independente. Os dados foram extraídos com base em um roteiro e apresentados no formato de fluxograma e quadros.

**Resultados:**

os 12 estudos selecionados nesta revisão mostraram que as características do cuidado de Enfermagem ampliado pela Antroposofia estão relacionadas ao conhecimento e habilidades para a aplicação de compressas, banhos, escalda-pés, deslizamento rítmico corporal, deslizamento corporal em pentagrama, cataplasma, administração de medicamentos, nos diferentes cenários de atenção à saúde, em condições agudas ou crônicas.

**Conclusão:**

o cuidado de enfermagem ampliado pela Antroposofia nos serviços de saúde é uma especialidade promissora no cenário mundial das práticas integrativas e complementares. Nessa direção, estudos futuros são necessários para a sua validação e valorização dos aspectos curativos do cuidado.Registro: https://doi.org/10.17605/OSF.IO/NV6D5

## Destaques

**(1)** Cuidados de Enfermagem Antroposófica (EA) integram-se com a enfermagem convencional. **(2)** Aplicabilidade dos cuidados de EA a diferentes contextos clínicos e de saúde. **(3)** A necessidade de conhecimento e habilidades advindos da Medicina Antroposófica. **(4)** A valoração dos aspectos curativos do cuidado implícitos na atitude do enfermeiro. **(5)** O potencial de atuação no cenário de saúde mundial e critérios de referência OMS.

## Introdução

Os cuidados de Enfermagem Antroposófica (EA) integram o sistema médico da Medicina Antroposófica (MA) e baseiam-se nas abordagens e competências convencionais da enfermagem, ampliadas pelo conhecimento antroposófico do ser humano. A diferenciação antroposófica entre corpo físico, forças vitais, alma e espírito, e a compreensão do desenvolvimento biográfico ao longo do percurso de vida proporcionam perspectivas adicionais sobre competências, processos e atitudes de enfermagem^1^ ^-^ ^2^. 

A MA é um sistema médico integrativo que amplia a medicina convencional com métodos cognitivos e conceitos da antroposofia estabelecidos por Rudolf Steiner e pela médica Ita Wegman, em 1920. Nesse sistema, as interações de vida expressas pelo corpo, alma e espírito são reguladas de acordo com a biografia, o contexto social e ambiental de cada pessoa, e podem ser amparadas com tratamentos e terapias por meio de medicamentos, cuidados de enfermagem, psicoterapia, arteterapia, euritmia terapêutica, terapias de movimento e corporais. Os tratamentos e terapias ofertados pela MA são direcionados ao indivíduo, à família, à comunidade e aos cuidadores, em consonância com as necessidades de saúde apresentadas nos contextos dos serviços de saúde. Destaca-se, nesse sistema, a possibilidade de tornar a compreensão espiritual do ser humano verificável e documentável, por meio da avaliação e articulação de aspectos não somente do corpo, como também da alma e do espírito^3^ ^-^ ^4^.

O ser humano, segundo a antroposofia, tem uma natureza quádrupla - corpo físico, etérico ou vital, corpo astral ou anímico, e espiritual ou “Eu”. O corpo físico é tudo o que pode ser tocado e medido, como sinais vitais, resultados laboratoriais, altura e peso. O corpo vital está ativo em processos e ritmos regenerativos do corpo, como digestão e sono. O corpo astral (alma) tem uma forte relação com pensamentos e emoções. Finalmente, o “Eu” do ser humano se manifesta na biografia, nos caminhos de vida, no destino e nas buscas espirituais como aspecto consciente do Eu^4^.

A prática da MA é realizada em ambientes de cuidados primários, secundários e terciários, centros de reabilitação, lares de idosos, serviços de enfermagem comunitários, hospitais e clínicas antroposóficas, hospitais públicos e centros médicos universitários. A abordagem é aplicada de forma integrada com a medicina ocidental convencional e em conjunto com outras especialidades médicas^1^ ^-^ ^2^. 

A equipe de enfermagem Antroposófica, em parceria com as do cuidado em MA, tem o mais alto nível de penetração da medicina integrativa na Alemanha e na Suíça, por meio da oferta de terapias nos hospitais antroposóficos e hospitais universitários acadêmicos, sendo que a sua prática é reconhecida pelos Conselhos Alemão e Holandês de Enfermagem^1^ ^,^ ^3^. 

No Brasil, a Medicina Antroposófica faz parte da lista de Práticas Integrativas e Complementares (PICs) institucionalizadas no Sistema Único de Saúde (SUS), pela Política Nacional de Práticas Integrativas e Complementares (PNPIC). A Antroposofia aplicada à saúde foi reconhecida há pouco tempo pelo Conselho Federal de Enfermagem como área de competência e atuação da enfermagem^5^ ^-^ ^6^. Recentemente, a EA passou a integrar os critérios (*Benchmarks*) da Organização Mundial da Saúde (OMS) para Medicina Antroposófica, que expõem normas e critérios para a formação e a prática dos profissionais de saúde^1^. 

À medida que países estabelecem estruturas regulatórias para a prática de abordagens de Terapias Integrativas e Complementares, os formuladores de políticas precisam de informações para a tomada de decisões, incluindo a avaliação da qualidade das práticas, dificuldades e formas de implementação^1^. 

Assim sendo, após busca preliminar na MEDLINE (via *PubMed*), JBI *Evidence Syntheses* e *Cochrane Database of Systematic Review*, não foram identificadas revisões sistemáticas ou de escopo, publicadas ou em curso, que investigaram as características do cuidado de enfermagem ampliado pela Antroposofia e suas práticas. Diante disso, este estudo tem como objetivo mapear as características do cuidado de enfermagem ampliado pela Antroposofia nos serviços de saúde^7^ ^-^ ^9^.

## Método

### Desenho do estudo

Trata-se de uma revisão de escopo orientada pelo JBI e o PRISMA *Extension for Scoping Reviews* (PRISMA-ScR): *Checklist and Explanation* ^7^ ^-^ ^9^. Para registro de intenção de desenvolvimento e disponibilidade científica, o protocolo foi registrado na plataforma *Open Science Framework* e pode ser acessado através do DOI 10.17605/OSF.IO/NV6D5^8^. Esta revisão objetivou responder à pergunta norteadora: “Quais as evidências na literatura sobre as características do cuidado de enfermagem ampliado pela Antroposofia nos serviços de saúde?” A elaboração desta pergunta foi feita por meio da utilização do acrônimo PCC, sendo a população (P) pessoas em qualquer fase do ciclo de vida humana, no que concerne ao conceito (C), características do cuidado de enfermagem ampliado pela Antroposofia e quanto ao contexto (C), cenários de atenção à saúde.

### Período

As buscas dos dados ocorreram entre os meses de março e abril de 2024.

### Critérios de seleção

Os critérios de inclusão aplicados foram: artigos originais e literatura cinzenta publicados sem restrição ao ano de publicação ou idioma. Foram excluídos artigos não disponíveis na íntegra, cujos títulos e resumos não respondiam à questão norteadora, além de cartas ao editor, editoriais, artigos de revisão, livros/capítulos de livros, e resumos de conferências.

### Estratégia de busca

Quanto à estratégia de pesquisa e identificação dos estudos, utilizaram-se as seguintes fontes de informações eletrônicas: *Academic Archive Online* (DiVA), *Academic Search Premier* (EBSCOhost), CINAHL *with full text* (via EBSCOhost), *Der Merkurstab*, EMBASE (Elsevier), LILACS (BVS), MEDLINE (via PubMed), MOSAICO (Modelos de Saúde e Medicinas Tradicionais, Integrativas e Complementares nas Américas- via BVS), SciELO (*Scientific Electronic Library Online*), Scopus (Elsevier), *ScienceDirect* (Elsevier), *Web of Science* - *Core Collection* (*Clarivate Analytics*). O acesso à revista *Der Merkurstab* ocorreu pelo *site Anthromedics*, ambiente virtual de referência em publicações na área de Medicina Antroposófica.

A busca da literatura cinzenta (dissertações e teses) foi conduzida nas plataformas da Biblioteca Digital Brasileira de Teses e Dissertações (BDTD), Catálogo de Teses e Dissertações da Coordenação de Aperfeiçoamento de Pessoal de Nível Superior (CAPES), *CyberTesis*, NDLTD (*Global ETD Search*), Repositório Científico de Acesso Aberto de Portugal (RCAAP), e *Theses Canada*.

A estratégia de busca foi elaborada com o apoio de um bibliotecário, utilizando os operadores booleanos AND e OR nos idiomas português, inglês e espanhol. Foram testadas diversas combinações de descritores extraídos do *Medical Subject Headings* (MeSH) e Descritores em Ciências da Saúde (DeCS). Esses descritores foram combinados em uma estratégia de pesquisa adaptada às particularidades de cada fonte de informação/repositório consultado na revisão, conforme ilustrado na Figura 1. O levantamento das publicações nas fontes de informações mencionadas ocorreu em 09 de abril de 2024.

**Figura 1:** Estratégias de busca desenvolvidas, adaptadas a cada fonte de dados. Brasília, DF, Brasil, 2024

**Fonte de dados**	**Estratégia de busca**
*Academic Archive Online* **(DiVA)**	Anthroposop* AND Nursing
*Academic Search Premier* **(EBSCOhost)**	(SU “Anthroposop*”) AND (SU “Nursing Care” OR “Nursing”) AND Health Care
Biblioteca Digital Brasileira de Teses e Dissertações (BDTD)	‘Anthroposop* AND (“Nursing Care” OR “Nursing”)’
Catálogo de Teses e Dissertações CAPES	Antroposofia e Enfermagem
*CyberTesis*	“Anthroposophical” OR “Anthroposophic” OR “Anthroposophy” OR “Antroposofia”
CINAHL *with full text* (via EBSCOhost)	(TI “Anthroposop*”) AND (“Nursing Care” OR “Nursing”) AND Health Care
*Der Merkurstab* **(via** *Anthromedics* **)**	Nursing
EMBASE (Elsevier)	‘anthroposop*’ AND (‘nursing care’/exp OR ‘nursing care’ OR ‘nursing’/exp OR ‘nursing’) AND (‘health care’/exp OR ‘health care’ OR ((‘health’/exp OR health) AND (‘care’/exp OR care)))
LILACS (BVS)	(db:”LILACS”) AND (Anthroposop*) AND (Nursing)
MEDLINE (via *PubMed*)	(“Antroposofia”[Título/Resumo] OR “Antroposop*”[Título/Resumo]) AND (“Cuidados de Enfermagem”[Título/Resumo] OR “Atencion de Enfermeria”[Título/Resumo] OR “Cuidados de Saúde”[Título/ Resumo] OU “Enfermagem”[Título/Resumo])
MOSAICO (Modelos de Saúde e Medicinas Tradicionais,Complementares, e Integrativas nas Américas - via BVS)	(anthroposop*) AND (nursing) AND ( collection_mtc:(“MTYCI”))
NDLTD (*Global ETD Search*)	“Anthroposophical” OR “Anthroposophic” OR “Anthroposophy” AND Nursing
Repositório Científico de Acesso Aberto de Portugal (RCAAP)	Anthroposophical OR Anthroposophic OR Anthroposophy AND Nursing
SciELO	(Anthroposophical OR Anthroposophic OR Anthroposophy ) AND (Nursing)
*Scopus* **(Elsevier)**	“Anthroposop*” AND ( “Nursing Care” OR “Nursing” ) AND health AND care
*ScienceDirect* **(Elsevier)**	(“Anthroposophical” OR “Anthroposophic” OR “Anthroposophy”) AND (“Nursing Care” OR “Nursing”)
*Theses Canada*	Anthroposop* AND Nursing
*Web of Science* **-** *Core Collection* **(** *Clarivate Analytics* **)**	“Anthroposop*” (Topic) AND (“Nursing Care” OR “Nursing”) (All Fields) AND Health Care (All Fields)

### Seleção de estudos

Após a busca, os estudos foram direcionados ao gerenciador *EndNoteWeb* para arquivos e exclusão de artigos em duplicidade. Em segundo momento, as referências foram enviadas ao *software Rayyan* ^*®*^ -*Intelligent Systematic Review* ^10^, acessado por dois revisores para leitura de títulos e resumos, e então os estudos foram classificados utilizando-se os critérios de inclusão e exclusão. Em caso de divergência, um terceiro revisor foi consultado. Na etapa seguinte, ocorreu a leitura dos estudos selecionados na íntegra e nova triagem utilizando os critérios de inclusão e exclusão. 

### Extração e apresentação de dados

A extração dos dados ocorreu por meio de um formulário de extração baseado nas recomendações do JBI^11^, adaptado pelos autores com as informações de interesse para esta revisão, a saber: a) Informações bibliográficas e Características do estudo: título, autor(es), ano, local de estudo (país), objetivo geral, tipo de estudo; b) Características do cuidado de enfermagem ampliado pela Antroposofia: características das intervenções de Enfermagem realizadas nos ambientes de cuidado (tipos, duração, frequência), serviço de saúde em que as intervenções de enfermagem são implementadas, resultados e recomendações.

A síntese dos resultados foi ilustrada no formato de fluxograma e quadros, sucedidos por um resumo narrativo dos resultados mapeados, descrevendo-os como esses estão relacionados com o objetivo e a pergunta da pesquisa. O estudo e a qualidade metodológica não foram avaliados, visto que o objetivo desta revisão de escopo não foi fornecer uma análise crítica das evidências. 

### Resultados

Após análise pareada e seleção dupla cega por revisores, ao final 12 publicações foram incluídas para revisão conforme Figura 2.

A Figura 3 apresenta os estudos selecionados para revisão de escopo, segundo informações bibliográficas e características do estudo.

**Figura 2:** Fluxograma de seleção dos estudos segundo recomendações PRISMA. Brasília, DF, Brasil, 2024

**Figura 3:** Caracterização das publicações mapeadas segundo autores, título do artigo, país e ano, tipo de estudo, amostra, objetivo e serviço de Saúde. Brasília, DF, Brasil, 2024

Código do estudo	Autores, título traduzido do artigo, país e ano	Tipo de estudo e amostra	Objetivo	Serviço de saúde
E1^12^	Bräuner G, Gerber W, Schulmayer H. Tratamento hospitalar de uma menina de 7 anos com constipação crônica e epilepsia. Apresentação de caso sob a perspectiva da terapia eurítmica, da enfermagem e da medicina, Alemanha 2004.	Estudo de caso. Menina de 7 anos com constipação crônica e convulsões.	Descrever o tratamento de uma menina de 7 anos com constipação crônica e epilepsia sob a perspectiva da euritmia terapêutica, da enfermagem e da medicina.	Hospital Antroposófico
E2^13^	Dach C, Heine R, Heiligtag HR. Cuidados antroposóficos para pacientes com câncer, Alemanha 2009.	Estudo de caso. Paciente do sexo feminino com diagnóstico de câncer de mama ductal invasivo no lado esquerdo, submetida a mastectomia conservadora e linfadenectomia.	Descrever as terapias de enfermagem no tratamento de pacientes com câncer em todas as fases da enfermidade.	Clínica Antroposófica
E3^14^	Stüdemann G. Compressas de borago para linfedema. Observações de uma perspectiva de enfermagem, Alemanha 2011.	Pesquisa qualitativa por meio de questionário. Dezessete pacientes oncológicos com linfedema.	Identificar a percepção de bem-estar de pacientes com linfedema, após tratamento com compressa de *Borago officinalis*.	Hospital Antroposófico
E4^15^	Ribeiro RM. O cuidado ampliado pela antroposofia: um estudo de caso sobre a prática da enfermagem Antroposófica, Brasil 2013.	Estudo de caso com observação participante. Equipe de enfermagem (1 enfermeira e 1 técnica de enfermagem).	Descrever o exercício da Enfermagem Antroposófica a partir de sua prática e vivência laboral em uma clínica médica antroposófica; identificar os tratamentos adotados no cuidado de Enfermagem Antroposófica; analisar os fundamentos do cuidado exercido pela enfermagem no ser humano.	Clínica Antroposófica
E5^16^	Therkleson T. Tratamento tópico de gengibre com compressa ou adesivo para os sintomas da osteoartrite, Nova Zelândia 2014.	Estudo randomizado. Vinte adultos com osteoartrite crônica.	Avaliar e comparar a percepção dos participantes acerca da intensidade da dor, fadiga crônica, efeito global da osteoartrite, mobilidade e satisfação com a saúde; uso de medicação para dor e/ou controle da dor; segurança da aplicação do adesivo e da compressa de gengibre.	Clínica Antroposófica e domicílio
E6^17^	Therkleson T. Terapia com gengibre para osteoartrite: um caso típico, Nova Zelândia 2014.	Estudo de caso. Paciente com osteoartrite crônica.	Descrever tratamento para osteoartrite com terapia do gengibre, aplicado por enfermeiros antroposóficos para um tipo específico de personalidade.	Clínica Antroposófica
E7^18^	Therkleson T. A busca de uma criança de 12 anos por si mesma: relato de caso, Nova Zelândia 2014.	Estudo de caso. Menino de 12 anos com transtorno de estresse pós-traumático complexo (C-PTSD).	Descrever os efeitos do tratamento de enfermagem antroposófica em um menino com estresse pós-traumático complexo.	Clínica Antroposófica
E8^19^	Kusserow M. Aplicações externas em práticas e clínicas antroposóficas, Alemanha 2014.	Estudo de caso. Menino de 5 anos com pneumonia bilateral.	Descrever o tratamento antroposófico para um menino de 5 anos com pneumonia bilateral viral.	Unidade de Homeopatia de um Hospital Regional
E9^20^	Therkleson T, Stronach S. Síndrome do coração partido: um caso típico, Nova Zelândia 2015.	Estudo de caso. Mulher de 82 anos com “Síndrome do Coração Partido”, exaustão geral e ataques de ansiedade.	Descrever tratamento para cardiomiopatia de Takotsubo “Síndrome do Coração Partido”.	Centro Médico e domicílio
E10^21^	Ström M. Deslizamento Rítmico segundo Wegman/Hauschka - um estudo de entrevista sobre as experiências dos pacientes com a terapia do toque em atendimento ambulatorial, Suécia 2016.	Análise de conteúdo. Cinco pacientes maiores de 18 anos.	Esclarecer como os pacientes que receberam deslizamento rítmico em atendimento ambulatorial vivenciaram o tratamento, e como os afetou.	Ambulatório Antroposófico
E11^22^	Deckers B, Schoen-Angerer T, Voggenreiter B, Vagedes J. Aplicações externas de enfermagem no gerenciamento de suporte do íleo pós-operatório prolongado: descrição das intervenções e relato de caso, Alemanha 2016.	Relato de caso. Paciente do sexo masculino de 61 anos com íleo pós-operatório prolongado.	Descrever a abordagem antroposófica da enfermagem para o tratamento de íleo pós-operatório prolongado.	Hospital Antroposófico
E12^23^	Fujiwara-Pichler E, Beckmann U, Madeleyn R. Pneumonia bacteriana adquirida na comunidade em criança tratada sem antibióticos em hospital de medicina antroposófica: relato de caso, Alemanha 2020.	Estudo de caso. Menino caucasiano de 4 ½ ano de idade com pneumonia bacteriana adquirida na comunidade (PAC), precedida por infecção mista de garganta viral e estreptocócica.	Descrever o tratamento de pneumonia bacteriana adquirida na comunidade em uma criança tratada sem antibióticos em um hospital de medicina antroposófica.	Hospital Antroposófico

Os cuidados de enfermagem ampliados pela antroposofia estão caracterizados na Figura 4, por meio da visualização do tipo, duração e frequência, resultados e recomendações descritos nos estudos. 

**Figura 4:** Caracterização das intervenções de enfermagem segundo tipo, duração, frequência, resultados e recomendações. Brasília, DF, Brasil, 2024

Código do estudo	Dado clínico	Tipo, duração e frequência dos cuidados de enfermagem	Resultados e recomendações
E1^12^	Menina de 7 anos com constipação crônica e convulsões.	Deslizamento abdominal com pomada *Oxalis folium* 10% em círculo ao redor do umbigo por cerca de 5 minutos; banho de dispersão de óleo de lavanda a 10% com descanso de 10 minutos após banho; deslizamento com óleo de lavanda a 10% nas pernas e pés com 10 minutos de descanso. Os banhos de dispersão realizaram-se em dias alternados; o deslizamento abdominal foi aplicado todos os dias antes do almoço; e o deslizamento nas pernas e pés todos os dias durante o período de internação.	Os cuidados de enfermagem combinados com arteterapia, massagem rítmica, elementos de educação curativa, conversa orientada para a situação famíliar e tratamento médico permitiu que após três semanas de internação, a constipação fosse curada completamente, com melhora das convulsões e harmonização sobre a situação familiar.
E2^13^	Mulher com câncer de mama submetida a mastectomia conservadora e linfadenectomia.	Na primeira semana de internação foi realizado: deslizamento rítmico nas costas com óleo de *Solum ulioginosum*; compressa no fígado com chá de *Achillea millefolium* após o almoço; envoltório no braço esquerdo com óleo de *Aconitum*; compressa de arnica em ambas as mamas para aliviar tensão, dor e sensação de peso nas mamas. Na segunda semana de internação: deslizamento rítmico nas costas e envoltório de queijo Quark em região de mama e tórax esquerdo. Os gestos de enfermagem antroposófica identificados nas primeiras duas semanas foram aliviar, envolver, confortar; nas semanas seguintes foram criar espaço e aliviar.	Paciente apresentou melhora da dor em braço, na mama, nas costas, e melhora da sensação de tensão e peso no braço e mama esquerda, sem necessidade de analgésicos e remédios para dormir no momento da alta clínica. Ensinada a realização de compressas e envoltórios para execução em domicílio para dor na região cervical. Recomenda-se as terapias externas antroposóficas como um complemento e uma extensão da assistência oncológica.
E3^14^	Paciente oncológico com linfedema.	Aplicação de compressa com Essência de *Borago*o*fficinalis L.* 20% durante 30 minutos no membro afetado, e após aplicação de pomada *Angelica archangelica* nas extremidades congestionadas, uma vez ao dia, durante 2 a 3 semanas ou até redução do edema.	A aplicação de compressas e pomadas trouxe alívio significativo dos sintomas advindos do linfedema, assim como, a percepção corporal de estar “mais leve”, “relaxado”, “perna mais magra”, e “revigorado”.
E4^15^	Atuação de enfermagem em clínica Antroposófica.	Realização de banho intestinal com chá composto de ervas, bicarbonato de sódio, sal grosso, sílica e fórmica; banho de dispersão de óleo; compressa nos órgãos com soluções e chás; escalda-pés com chás, tinturas e sais; deslizamento corporal; envoltório de todo o corpo com lençol aquecido; administração parenteral de medicamentos. O tempo de aplicação das terapias variaram conforme terapêutica aplicada, em média de trinta a sessenta minutos. Após a aplicação da terapia o paciente permanecia num ambiente de repouso e silêncio para que ocorresse a fase regenerativa. A frequência das intervenções era diversificada, direcionada pela prescrição médica.	As terapias identificadas foram o banho intestinal, compressas, deslizamento corporal, banhos de imersão com óleo, envoltórios, escalda-pés, e administração da terapia com *Viscum album* em pacientes oncológicos. O cuidado foi fundamentado nos doze sentidos, nos sete processos vitais, nos doze gestos do cuidado, na trimembração e na quadrimembração. Destaca o paciente como foco de cuidado, e que o processo de cura envolve a existência de forças sutis que podem ser estimuladas pelo cuidado de enfermagem.
E5^16^	Pacientes adultos com osteoartrite crônica.	Aplicação de adesivo de matéria orgânica seca moída de rizoma *Zingiber officinale*, ou compressa de gengibre na região médio-lombar por 30 minutos, seguido de um descanso de 20 minutos. Aplicação tópica uma vez ao dia de adesivo ou compressa de gengibre por 7 dias consecutivos; auto aplicação tópica de adesivo de gengibre em domicílio por 24 semanas.	Após 1 semana de tratamento tópico com gengibre houve declínio nas pontuações em dor, fadiga, efeito global e estado funcional, enquanto a satisfação com a saúde melhorou de 80% de insatisfação para 70% satisfeito. As pontuações em todos os cinco domínios foram reduzidas progressivamente ao longo do após 24 semanas de auto tratamento, assim como a redução do uso de analgésicos chegando ao não uso de medicação para dor pela maioria dos participantes ao final do período de estudo. Recomenda-se o tratamento tópico com gengibre, pois sua aplicação é simples e econômica, devendo ser considerada no cuidado de idosos com osteoartrite crônica.
E6^17^	Paciente com osteoartrite.	Aplicação de compressa de gengibre na região médio-lombar (renal) por 30 minutos, seguido de um descanso de 15 minutos, por 7 dias consecutivos, e aplicações contínuas por duas ou três vezes por semana, para manutenção dos níveis de dor.	A compressa de gengibre trouxe alívio imediato e progressivo dos sintomas de osteoartrite ao longo de 24 semanas, sem efeitos negativos relatados pelo paciente. Recomenda-se o uso da compressa de gengibre no cuidado de enfermagem a pacientes com osteoartrite.
E7^18^	Menino de 12 anos com transtorno de estresse pós-traumático complexo.	Terapia combinada de escalda pés com limão, deslizamento rítmico corporal com óleo *Solum ulioginosum*, deslizamento cardíaco com *Aurum*-Lavanda-Rosa pomada; deslizamento em pentagrama com pomada *Aurum*-Lavanda-Rosa. As terapias foram aplicadas uma vez por semana, durante 5 semanas, em sessões de 60 minutos, com 15 minutos de descanso entre as terapias.	O escalda-pés, massagem cardíaca e deslizamentos rítmicos, indicados para os sintomas de transtorno de estresse pós-traumático complexo, somente surtiram efeito após a inclusão de deslizamento em pentagrama. Recomenda-se que o deslizamento em pentagrama seja considerado no gerenciamento de trauma, estresse, exaustão e desconexão em crianças de 12 anos.
E8^19^	Menino de 5 anos com pneumonia bilateral.	Aplicação de gel de *Cochlearia armoracia* nos seios nasais; compressa de *Achillea millefolium*, com *Petacites officinalis* em região torácica, por 45 minutos, uma vez ao dia, por 14 dias. Compressa de limão em momentos de febre.	As compressas, trouxeram alívio dos sintomas de tosse e esforço para respirar, relaxamento para dormir, recuperação do apetite, melhora do estado de ânimo - se pondo mais contente, e autorregulação da temperatura.
E9^20^	Mulher de 82 anos com “Síndrome do Coração Partido”.	Sessões de escalda-pés com lavanda, deslizamento rítmico usando óleo *Solum ulioginosum* e cataplasma quente de pomada *Oxalis folium*, uma sessão por semana, durante 4 semanas. Em domicílio, durante o tratamento o paciente recebeu uma “almofada cardíaca” impregnada com pomada de *Aurum* Lavanda-Rosa, para ser aplicada sobre o seio esquerdo à noite, uso diário por 4 semanas.	O tratamento com escalda-pés e deslizamento rítmico não interferiu na resposta aos medicamentos em uso, e permitiu que a idosa traumatizada pudesse superar o medo e encontrar coragem renovada para responder a seu mundo e ao dos outros. Recomenda-se o uso de escalda-pés e deslizamento rítmico quando há opções limitadas para gerenciar traumas e estresse.
E10^21^	Pacientes que receberam deslizamento rítmico.	Sessões de deslizamento rítmico em ambiente ambulatorial, aplicada 1 ou 2 vezes por semana, durante 4 a 8 semanas.	O deslizamento rítmico trouxe melhora da função pulmonar (tosse, asma e doença pulmonar obstrutiva crônica), função intestinal, dor de cabeça, dor geral em condições de dor prolongada, e sono. Experimentar o cuidado oferecido foi descrito como sentir estabilidade, segurança, sentimento de conexão e relaxamento. A experiência de ser confirmado foi descrita como recuperar os limites, ser visto, sentir-se, sentir significado e participante de um contexto. A vitalidade foi expressa como experiências de se sentir mais forte, com esperança e com mais energia. Experiências de saúde apesar do sofrimento e doença grave foi descrita como sentir-se bem e calmo em uma situação de vida caótica.
E11^22^	Paciente com íleo pós-operatório prolongado.	Aplicação de compressa com tintura de *Oxalis folium* 20% na região abdominal durante 20 minutos, seguido por 30 minutos de descanso; realização de massagem com óleo de melissa no abdômen, no sentido horário, por cerca de 5 minutos; aplicação de compressa com pomada *Thuja occidentalis* 10% e *Argentum Metallicum* 0,4%, em quantidades iguais, no abdômen por 30 minutos, seguido de 30 minutos de descanso. Durante 10 dias, 1 vez pela manhã, foi aplicada compressa de *Oxalis folium* seguida de massagem com óleo de melissa; e 1 vez à noite, compressa de pomada *Thuja*/*Argentum*.	O uso de compressas e massagens abdominais melhorou a sensação de bem-estar e redução da dor, sendo possível gerir o íleo pós-operatório prolongado sem drogas procinéticas. Recomenda-se que compressas e massagem leve em região abdominal sejam utilizadas como ferramentas adicionais no manejo do íleo pós-operatório prolongado.
E12^23^	Menino de 4 ½ anos de idade com pneumonia bacteriana adquirida na comunidade.	Aplicação de compressa morna de queijo tipo Quark na parede torácica direita, por 20 minutos no 1º dia de tratamento; aplicação de compressa de óleo de lavanda morna na parede torácica anterior, uma vez ao dia durante 6 dias; aplicação de bolsa de água quente para hipotermia após medicamento retal para febre; do 2º ao 6º dia de tratamento recebeu compressa de mostarda moída na parede torácica direita, por 2 a 3 minutos.	A pneumonia foi tratada de forma eficaz e segura, sem o uso de antibióticos, mediante pediatras preparados para reavaliar a criança com frequência, equipe de enfermagem familiarizada com esse tratamento, e apoio dos pais. Recomenda-se que as terapias sejam administradas em instalações apropriadas e especializadas para este tipo de tratamento, incluindo pessoal altamente treinado para reduzir o risco de complicações, incluindo a morte.

## Discussão

Dentre os estudos analisados nesta revisão, no que se refere ao idioma, seis foram redigidos em língua inglesa, quatro em alemã, um em português e um em sueco. Em relação ao tipo de publicação, dez correspondem a artigos científicos, um a dissertação de mestrado e um a tese de doutorado. Quanto à origem geográfica, quatro estudos são provenientes da Nova Zelândia, seis da Alemanha, um do Brasil e um da Suécia^12^ ^-^ ^23^.

Esta revisão possibilitou mapear as características do cuidado ampliado pela antroposofia, evidenciando que a EA se utiliza das terapias externas, expressa nas práticas de compressas, cataplasmas, envoltórios, banhos, escalda-pés, lavagens intestinais, massagem com mel, deslizamentos rítmicos, pentagrama, administração parenteral e inalatória de medicamentos, usadas para queixas físicas (dor, tosse, asma, constipação, dificuldade para respirar, convulsões, tensão muscular, fadiga, falta de apetite, dificuldade de autorregulação da temperatura, pneumonia) e anímicas (medo, sofrimento psíquico, insatisfação com a saúde, estresse, exaustão mental, humor deprimido). Essas terapias combinam atenção interpessoal, toque, movimentos rítmicos, manutenção de calor, elementos fitoterápicos (óleo, essências, tinturas, pomadas) em intervenções aplicadas diariamente ou 1-2 vezes por semana, em sessões de 30-60 minutos, com duração a depender da substância aplicada, que são seguidas por um período de repouso terapêutico, e administradas em ciclos de tratamentos de acordo com a condição clínica do paciente^12^ ^-^ ^23^.

Nos estudos analisados, as terapias externas foram associadas à terapia medicamentosa antroposófica no início do tratamento, visando estimular a autorregulação do calor corporal e mobilização das forças etéricas, sendo o veículo para acesso dos medicamentos aos outros corpos, anímico e eu, contribuindo para que a medicação oral surta efeito mais rápido, e mobilize forças que auxiliam no processo de cura^12^ ^,^ ^14^ ^,^ ^16^ ^,^ ^18^ ^-^ ^20^ ^,^ ^22^ ^-^ ^23^. Primeiramente as terapias externas são coadjuvantes no processo terapêutico, mas, com o passar das sessões, tornam-se a principal terapia curativa^13^ ^,^ ^16^ ^-^ ^17^. As terapias externas são apontadas na literatura como facilitadoras da autorregulação autonômica e de processos salutogênicos (que promovem e potencializam o desenvolvimento de um bom estado subjetivo de saúde), influenciando fisicamente a distribuição de calor no organismo, integrando as funções do corpo, alma e espírito, por meio da regulação de processos vitais, como respiração, aquecimento, nutrição, excreção, manutenção, crescimento e reprodução, assim como, da harmonização das questões emocionais, e de processos de significação da vida e da saúde^2^ ^,^ ^24^.

Os estudos apontam o indivíduo como foco de cuidado na EA, mediante o reconhecimento de sua natureza quádrupla^12^ ^-^ ^23^. A literatura explica que, ao observar como os aspectos dessa natureza quádrupla interagem entre si e como são expressados, um novo nível de compreensão é alcançado e ocorre um processo de avaliação empírica dos corpos mais sutis. Por exemplo, a funcionalidade do corpo vital, que mantém todas as partes numa relação viva, pode ser vista no equilíbrio de fluidos, nos ciclos de sono/vigília e na qualidade do cabelo e das unhas. A saúde do corpo emocional pode ser avaliada pelo equilíbrio emocional, experiências de dor e fortes reações às circunstâncias ou pessoas com simpatia ou antipatia. Por fim, a presença do “eu” como ativo no indivíduo pode ser observada através do senso de coerência que se tem na vida, da conexão com a espiritualidade e/ou do mundo, da capacidade de se tornar observador tanto dos outros quanto de si mesmo, e a capacidade de encontrar significado na vida ou na doença. Isso proporciona ao enfermeiro antroposófico uma compreensão profunda dos outros e de si mesmo, e naturalmente passa a informar o cuidado prestado^4^ ^,^ ^24^.

Os estudos destacaram que o cuidado de EA é ofertado a pacientes nos diferentes ciclos de vida, com condições clínicas diversas, agudas ou crônicas, com intervenções que produzem impactos imediatos e a curto prazo na saúde, em ambientes de atenção primária, atendimento ambulatorial e hospitalar e que podem estar integrados com práticas de saúde da medicina convencional^12^ ^-^ ^23^. Também foi realçada a tomada de decisão compartilhada e ênfase na autogestão de sintomas relacionados à doença, com resultados a médio e longo prazo, viabilizada pela possibilidade de algumas terapias externas serem ensinadas para auto aplicação em domicílio^13^ ^,^ ^16^ ^-^ ^17^ ^,^ ^20^, principalmente a pacientes que enfrentam sintomas crônicos, devido à natureza simples de execução e ao baixo custo de seus elementos terapêuticos.

Em outro âmbito os cuidados foram evidenciados, como nas doenças crônicas em crianças. Considerou-se que as doenças crônicas advêm do modo de vida atual e de experiências sensoriais e mentais na infância, e podem ser tratadas com a terapia antroposófica, principalmente quando outras terapias foram tentadas sem êxito. O objetivo é substituir influências que causam doenças por outras que preservam a saúde. A integração mútua de terapias de enfermagem, terapia artística, deslizamento rítmico, educação curativa, debates orientados com a família, e terapia farmacológica colaboram para que se crie um equilíbrio na percepção empática da carga familiar individual e uma identificação clara das condições e consequências das influências do cotidiano familiar na saúde das crianças. Neste contexto, se reconhece que por vezes o tratamento em ambiente ambulatorial não surte o efeito desejado, sendo necessário acompanhamento em ambiente hospitalar, para que os aspectos físicos e anímicos possam ser compreendidos e acompanhados com um plano terapêutico mais assertivo para a criança e o contexto familiar^12^.

O cuidado de EA na área de oncologia foi ressaltado por meio das terapias externas como compressas, envoltórios, deslizamentos rítmicos segundo Wegman e Hauschka, medicamentos antroposóficos, orientações de cuidados de saúde para domicílio, e atitude interior do Enfermeiro, que configuram um complemento à assistência oncológica. A terapêutica no contexto oncológico se baseou na promoção de calor interno, para que se estabeleça a integração entre os quatro corpos do ser humano e se estimule o princípio curativo. Destaca-se que as terapias externas ofertadas advêm da decisão do enfermeiro, embasado nos doze gestos da enfermagem antroposófica, que oferecem um modelo de avaliação, orientação e execução do plano terapêutico da enfermagem^13^ ^-^ ^14^.

Os aspectos curativos do cuidado de EA e o envolvimento do paciente são apontados na literatura como atividades intangíveis que contribuem para resultados e experiências positivas do paciente, e precisam ser valorados nos contextos de saúde em igual importância com os processos terapêuticos convencionais de cura^4^.

Os estudos destacaram que o nível de consciência que os enfermeiros compartilham em suas intervenções eleva o trabalho da enfermagem para além de modelos mecânicos, um nível terapêutico, que inclui as qualidades internas intangíveis que os enfermeiros trazem para a experiência do cuidado, ou seja, a ampliação da consciência não sobre o que a enfermeiro faz pelo paciente, mas como isso é feito. Esse processo possibilita ao enfermeiro compreender de que forma o cuidado oferecido afeta os quatro corpos do paciente, além de influenciar seu humor interno e o estado de ser do próprio profissional. Cada encontro é visto como único, carregando em si o potencial de transformação para todos os envolvidos no ato de cuidado. A intenção consciente pode ser exemplificada por atitudes expressas em 12 gestos da enfermagem, que fortalecem o enfermeiro e apoiam o paciente^13^ ^,^ ^15^ ^,^ ^19^ ^,^ ^21^. Os doze gestos de Enfermagem Antroposófica são classificados em dois grupos: gestos envolventes e gestos ativadores. Os gestos envolventes visam satisfazer uma necessidade da qual a pessoa, em determinada condição, não é capaz de suprir autonomamente. Nessa abordagem, o enfermeiro atua de forma interventiva, em nome da segurança ou conforto do paciente. Já os gestos ativadores têm como objetivo estimular os pacientes a se tornarem conscientes e a agirem em seu próprio nome, favorecendo o resgate de sua capacidade interna de reação e autorregulação^4^.

Diante dos achados, a limitação deste estudo é seu caráter de escopo, explorando publicações que descrevessem os cuidados de enfermagem ampliados pela antroposofia, o que limitou a expressividade da revisão, pois algumas pesquisas enfocavam as terapias externas, mas com desenhos metodológicos pouco claros, que não permitiam identificar se a terapia referida era utilizada na prática de enfermagem. Recomenda-se que estudos futuros foquem em uma abordagem metodológica objetiva e clara, e ampliem o cenário de atuação da EA ao âmbito gerencial, de ensino e de especialidades na área de saúde, referenciadas na literatura, mas que não foram evidenciadas, como por exemplo a Enfermagem Obstétrica Antroposófica^1^.

Os resultados provenientes desta revisão contribuem para o avanço do conhecimento científico na área das PICs, especialmente para a enfermagem antroposófica. Essa área exige o aprimoramento de habilidades e conhecimentos especializados, para que os profissionais possam incorporar novas tecnologias e práticas, além de competências interpessoais essenciais para lidar com as diversas situações que a profissão apresenta. As evidências coletadas auxiliam no preenchimento da lacuna de conhecimento sobre o fazer da Enfermagem Antroposófica e destaca a necessidade de futuras pesquisas que consolidem esse conhecimento e ampliem as formas de cuidado de enfermagem em diferentes contextos de saúde.

## Conclusão

O mapeamento das características do cuidado de enfermagem ampliado pela Antroposofia nos serviços de saúde evidenciou a Enfermagem Antroposófica como especialidade na área de enfermagem, com um fazer baseado em conhecimento próprio advindo da Medicina Antroposófica, que possibilita a integração com práticas de saúde da medicina convencional e atuação em equipe multiprofissional. Apresenta o indivíduo como foco de cuidado, em diferentes contextos de saúde, seja hospitalar, de clínicas de saúde ou domicílio, com condições clínicas diversas, agudas ou crônicas.

Os cuidados de enfermagem requerem do enfermeiro conhecimentos e habilidades específicas sobre as práticas terapêuticas antroposóficas, para oferecer uma assistência eficaz e segura nos diferentes contextos de saúde e de vida. Essas práticas têm características de acompanhamento a curto, médio e longo prazo, sendo que algumas podem ser ensinadas ao paciente para realização em domicílio, como forma complementar ao plano terapêutico estabelecido. O destaque recai sobre a corresponsabilidade no processo terapêutico e na postura do enfermeiro percebida pelos pacientes como fator facilitador do processo de cura. A Enfermagem Antroposófica amplia o olhar sobre a promoção da saúde e necessidades individuais dos pacientes que, por vezes, estão além dos limites da medicina convencional.

## Declaração de Disponibilidade de Dados:

Todos os dados gerados ou analisados durante este estudo estão incluídos neste artigo publicado.

# Translation: Características del cuidado de enfermería ampliado por la antroposofía: revisión de alcance

**DOI:** 10.1590/1518-8345.7819.4791

**Authors:** Susana Martín-Hernández, Janaina Meirelles Sousa, Georgina Casanova-Garrigos

**Affiliations:** 1 Universitat Rovira i Virgili, Campus Terres de l’Ebre, Facultat d’Infermeria, Tortosa, CT, España.; 2 Universidade de Brasília, Campus Ceilândia, Faculdade de Ciências de Tecnologias em Saúde, Ceilândia, DF, Brasil.

**Keywords:** Atención a la Salud, Atención de Enfermería, Enfermería, Medicina Antroposófica, Servicios de Salud, Terapias Complementarias.

## Abstract

**Objetivo:**

mapear las características del cuidado de enfermería ampliado por la Antroposofía en los servicios de salud.

**Método:**

revisión de alcance orientada por el JBI. Se incluyeron 18 fuentes de información y se consideraron elegibles artículos originales y literatura gris, publicados sin restricción por año de publicación o idioma. Tras la eliminación de duplicados, la selección fue realizada por dos revisoras de forma ciega e independiente. Los datos fueron extraídos con base en un guion y presentados en formato de diagrama de flujo y cuadros.

**Resultados:**

los 12 estudios seleccionados en esta revisión mostraron que las características del cuidado de Enfermería ampliado por la Antroposofía están relacionadas con el conocimiento y las habilidades para la aplicación de compresas, baños, baños de pies, deslizamiento rítmico corporal, deslizamiento corporal en pentagrama, cataplasma, administración de medicamentos, en los diferentes escenarios de atención a la salud, en condiciones agudas o crónicas.

**Conclusión:**

el cuidado de enfermería ampliado por la Antroposofía en los servicios de salud es una especialidad prometedora en el escenario mundial de las prácticas integrativas y complementarias. En esta dirección, se necesitan estudios futuros para su validación y para la valorización de los aspectos curativos del cuidado. Registro: https://doi.org/10.17605/OSF.IO/NV6D5

## Destacados

**(1)** Los Cuidados de Enfermería Antroposófica (EA) se integran con la enfermería convencional. **(2)** Aplicabilidad de los cuidados de EA a distintos contextos clínicos y de salud. **(3)** La necesidad de conocimientos y habilidades provenientes de la Medicina Antroposófica. **(4)** La valoración de los aspectos curativos del cuidado implícitos en la actitud del profesional de enfermería. **(5)** El potencial de actuación en el escenario de salud mundial y criterios de referencia de la OMS.

## Introducción

Los cuidados de Enfermería Antroposófica (EA) integran el sistema médico de la Medicina Antroposófica (MA) y se basan en los abordajes y competencias convencionales de la enfermería, ampliados por el conocimiento antroposófico del ser humano. La diferenciación antroposófica entre cuerpo físico, fuerzas vitales, alma y espíritu, y la comprensión del desarrollo biográfico a lo largo del curso de la vida proporcionan perspectivas adicionales sobre competencias, procesos y actitudes de enfermería^1^ ^-^ ^2^. 

La MA es un sistema médico integrativo que amplía la medicina convencional con métodos cognitivos y conceptos de la antroposofía establecidos por Rudolf Steiner y la médica Ita Wegman, en 1920. En este sistema, las interacciones de vida expresadas por el cuerpo, el alma y el espíritu se regulan de acuerdo con la biografía, el contexto social y ambiental de cada persona, y pueden ser sustentadas con tratamientos y terapias mediante medicamentos, cuidados de enfermería, psicoterapia, arteterapia, euritmia terapéutica, terapias de movimiento y corporales. Los tratamientos y terapias ofrecidos por la MA se dirigen al individuo, a la familia, a la comunidad y a los cuidadores, en consonancia con las necesidades de salud presentadas en los contextos de los servicios de salud. Se destaca, en este sistema, la posibilidad de hacer verificable y documentable la comprensión espiritual del ser humano, mediante la evaluación y articulación de aspectos no solo del cuerpo, sino también del alma y del espíritu^3^ ^-^ ^4^.

El ser humano, según la antroposofía, tiene una naturaleza cuádruple: cuerpo físico, etérico o vital, cuerpo astral o anímico, y espiritual o “Yo”. El cuerpo físico es todo lo que puede ser tocado y medido, como signos vitales, resultados de laboratorio, altura y peso. El cuerpo vital está activo en procesos y ritmos regenerativos del cuerpo, como la digestión y el sueño. El cuerpo astral (alma) tiene una relación estrecha con los pensamientos y las emociones. Finalmente, el “Yo” del ser humano se manifiesta en la biografía, en los caminos de vida, en el destino y en las búsquedas espirituales como aspecto consciente del Yo^4^.

La práctica de la MA se realiza en entornos de cuidados primarios, secundarios y terciarios, centros de rehabilitación, residencias de personas mayores, servicios de enfermería comunitarios, hospitales y clínicas antroposóficas, hospitales públicos y centros médicos universitarios. El abordaje se aplica de forma integrada con la medicina occidental convencional y en conjunto con otras especialidades médicas^1^ ^-^ ^2^. 

El equipo de enfermería Antroposófica, en colaboración con los cuidados en MA, tiene el más alto nivel de penetración de la medicina integrativa en Alemania y Suiza, mediante la oferta de terapias en los hospitales antroposóficos y hospitales universitarios académicos, y su práctica está reconocida por los Consejos Alemán y Neerlandés de Enfermería^1^ ^,^ ^3^. 

En Brasil, la Medicina Antroposófica forma parte de la lista de Prácticas Integrativas y Complementarias institucionalizadas en el Sistema Único de Salud (SUS), por la Política Nacional de Prácticas Integrativas y Complementarias (PNPIC). La Antroposofía aplicada a la salud fue reconocida hace poco tiempo por el Consejo Federal de Enfermería como área de competencia y actuación de la enfermería^5^ ^-^ ^6^. Recientemente, la EA pasó a integrar los criterios (*Benchmarks*) de la Organización Mundial de la Salud (OMS) para la Medicina Antroposófica, que exponen normas y criterios para la formación y la práctica de los profesionales de la salud^1^. 

A medida que los países establecen estructuras regulatorias para la práctica de enfoques de Terapias Integrativas y Complementarias, los responsables de políticas necesitan información para la toma de decisiones, incluida la evaluación de la calidad de las prácticas, las dificultades y las formas de implementación^1^. 

Así, tras una búsqueda preliminar en MEDLINE (vía *PubMed*), JBI *Evidence Syntheses y Cochrane Database of Systematic Review*, no se identificaron revisiones sistemáticas o de alcance, publicadas o en curso, que investigaran las características del cuidado de enfermería ampliado por la Antroposofía y sus prácticas. Ante ello, este estudio tiene como objetivo mapear las características del cuidado de enfermería ampliado por la Antroposofía en los servicios de salud^7^ ^-^ ^9^.

## Método

### Diseño del estudio

Se trata de una revisión de alcance orientada por el JBI y el PRISMA *Extension for Scoping Reviews* (PRISMA-ScR): *Checklist and Explanation* ^7^ ^-^ ^9^. Para el registro de intención de desarrollo y disponibilidad científica, el protocolo fue registrado en la plataforma *Open Science Framework* y puede accederse a través del DOI 10.17605/OSF.IO/NV6D5^8^. Esta revisión tuvo como objetivo responder a la pregunta orientadora: “¿Cuáles son las evidencias en la literatura sobre las características del cuidado de enfermería ampliado por la Antroposofía en los servicios de salud?” La elaboración de esta pregunta se realizó mediante la utilización del acrónimo PCC, siendo la población (P) personas en cualquier fase del ciclo de vida humano; en lo que concierne al concepto (C), características del cuidado de enfermería ampliado por la Antroposofía; y en cuanto al contexto (C), escenarios de atención a la salud.

### Período

Las búsquedas de datos se realizaron entre los meses de marzo y abril de 2024.

### Criterios de selección 

Los criterios de inclusión aplicados fueron: artículos originales y literatura gris publicados sin restricción por año de publicación o idioma. Se excluyeron artículos no disponibles en texto completo, cuyos títulos y resúmenes no respondían a la pregunta orientadora, además de cartas al editor, editoriales, artículos de revisión, libros/capítulos de libros y resúmenes de conferencias.

### Estrategia de búsqueda

En cuanto a la estrategia de búsqueda e identificación de los estudios, se utilizaron las siguientes fuentes de información electrónicas: *Academic Archive Online* (DiVA), *Academic Search Premier* (EBSCOhost), CINAHL *with full text* (vía EBSCOhost), *Der Merkurstab*, EMBASE (Elsevier), LILACS (BVS), MEDLINE (vía PubMed), MOSAICO (Modelos de Salud y Medicinas Tradicionales, Integrativas y Complementarias en las Américas - vía BVS), SciELO (*Scientific Electronic Library Online*), Scopus (Elsevier), *ScienceDirect* (Elsevier), *Web of Science* - *Core Collection* (*Clarivate Analytics*). El acceso a la revista *Der Merkurstab* se realizó a través del sitio *Anthromedics*, entorno virtual de referencia en publicaciones en el área de Medicina Antroposófica.

La búsqueda de la literatura gris (disertaciones y tesis) fue conducida en las plataformas *Biblioteca Digital Brasileira de Teses e Dissertações* (BDTD), *Catálogo de Teses e Dissertações* de la *Coordenação de Aperfeiçoamento de Pessoal de Nível Superior* (CAPES), *CyberTesis*, NDLTD (*Global ETD Search*), *Repositório Científico de Acesso Aberto de Portugal* (RCAAP) y *Theses Canada*.

La estrategia de búsqueda fue elaborada con el apoyo de un bibliotecario, utilizando los operadores booleanos AND y OR en los idiomas portugués, inglés y español. Se probaron diversas combinaciones de descriptores extraídos de *Medical Subject Headings* (MeSH) y Descriptores en Ciencias de la Salud (DeCS). Estos descriptores fueron combinados en una estrategia de búsqueda adaptada a las particularidades de cada fuente de información/repositorio consultado en la revisión, conforme se ilustra en la Figura 1. El levantamiento de las publicaciones en las fuentes de información mencionadas tuvo lugar el 09 de abril de 2024.

**Figura 1:** Estrategias de búsqueda desarrolladas, adaptadas a cada fuente de datos. Brasilia, DF, Brasil, 2024

**Fuente de datos**	**Estrategia de búsqueda**
*Academic Archive Online* **(DiVA)**	Anthroposop* AND Nursing
*Academic Search Premier* **(EBSCOhost)**	(SU “Anthroposop*”) AND (SU “Nursing Care” OR “Nursing”) AND Health Care
*Biblioteca Digital Brasileira de Teses e Dissertações* **(BDTD)**	‘Anthroposop* AND (“Nursing Care” OR “Nursing”)’
*Catálogo de Teses e Dissertações* **CAPES**	Antroposofia e Enfermagem
*CyberTesis*	“Anthroposophical” OR “Anthroposophic” OR “Anthroposophy” OR “Antroposofia”
CINAHL *with full text* (via EBSCOhost)	(TI “Anthroposop*”) AND (“Nursing Care” OR “Nursing”) AND Health Care
*Der Merkurstab* **(via** *Anthromedics* **)**	Nursing
EMBASE (Elsevier)	‘anthroposop*’ AND (‘nursing care’/exp OR ‘nursing care’ OR ‘nursing’/exp OR ‘nursing’) AND (‘health care’/exp OR ‘health care’ OR ((‘health’/exp OR health) AND (‘care’/exp OR care)))
LILACS (BVS)	(db:”LILACS”) AND (Anthroposop*) AND (Nursing)
MEDLINE (via *PubMed*)	(“Antroposofia”[Título/Resumo] OR “Antroposop*”[Título/Resumo]) AND (“Cuidados de Enfermagem”[Título/Resumo] OR “Atencion de Enfermeria”[Título/Resumo] OR “Cuidados de Saúde”[Título/ Resumo] OU “Enfermagem”[Título/Resumo])
MOSAICO (Modelos de Salud y Medicinas Tradicionales, Integrativas y Complementarias en las América - vía BVS)	(anthroposop*) AND (nursing) AND ( collection_mtc:(“MTYCI”))
NDLTD (*Global ETD Search*)	“Anthroposophical” OR “Anthroposophic” OR “Anthroposophy” AND Nursing
*Repositório Científico de Acesso Aberto de Portugal* **(RCAAP)**	Anthroposophical OR Anthroposophic OR Anthroposophy AND Nursing
SciELO	(Anthroposophical OR Anthroposophic OR Anthroposophy ) AND (Nursing)
*Scopus* **(Elsevier)**	“Anthroposop*” AND ( “Nursing Care” OR “Nursing” ) AND health AND care
*ScienceDirect* **(Elsevier)**	(“Anthroposophical” OR “Anthroposophic” OR “Anthroposophy”) AND (“Nursing Care” OR “Nursing”)
*Theses Canada*	Anthroposop* AND Nursing
*Web of Science* **-** *Core Collection* **(** *Clarivate Analytics* **)**	“Anthroposop*” (Topic) AND (“Nursing Care” OR “Nursing”) (All Fields) AND Health Care (All Fields)

### Selección de estudios

Tras la búsqueda, los estudios fueron dirigidos al gestor *EndNoteWeb* para archivo y exclusión de artículos duplicados. En una segunda etapa, las referencias se enviaron al *software Rayyan* ^*®*^ -*Intelligent Systematic Review* ^10^, al que accedieron dos revisores para la lectura de títulos y resúmenes, y entonces los estudios fueron clasificados utilizando los criterios de inclusión y exclusión. En caso de discrepancia, se consultó a un tercer revisor. En la etapa siguiente, se procedió a la lectura íntegra de los estudios seleccionados y a un nuevo cribado utilizando los criterios de inclusión y exclusión.

### Extracción y presentación de datos

La extracción de los datos se realizó mediante un formulario de extracción basado en las recomendaciones del JBI^11^, adaptado por los autores con la información de interés para esta revisión, a saber: a) Información bibliográfica y Características del estudio: título, autor(es), año, lugar del estudio (país), objetivo general, tipo de estudio; b) Características del cuidado de enfermería ampliado por la Antroposofía: características de las intervenciones de Enfermería realizadas en los entornos de cuidado (tipos, duración, frecuencia), servicio de salud en el que se implementan las intervenciones de enfermería, resultados y recomendaciones.

La síntesis de los resultados se ilustró en formato de diagrama de flujo y cuadros, seguidos de un resumen narrativo de los resultados mapeados, describiéndolos en relación con el objetivo y la pregunta de investigación. El estudio y la calidad metodológica no fueron evaluados, dado que el objetivo de esta revisión de alcance no fue proporcionar un análisis crítico de las evidencias. 

## Resultados

Tras el análisis en pares y la selección a doble ciego por revisores, al final se incluyeron 12 publicaciones para la revisión, conforme a la Figura 2.

La Figura 3 presenta los estudios seleccionados para la revisión de alcance, según la información bibliográfica y las características del estudio.

**Figura 2:** Diagrama de flujo de selección de los estudios según las recomendaciones PRISMA. Brasilia, DF, Brasil, 2024

**Figura 3:** Caracterización de las publicaciones mapeadas según autores, título del artículo, país y año, tipo de estudio, muestra, objetivo y servicio de salud. Brasilia, DF, Brasil, 2024

Código del estudio	Autores, título traducido del artículo, país y año	Tipo de estudio y muestra	Objetivo	Servicio de salud
E1^12^	Bräuner G, Gerber W, Schulmayer H. Tratamiento hospitalario de una niña de 7 años con estreñimiento crónico y epilepsia. Presentación del caso desde una perspectiva de euritmia terapéutica, enfermería y medicina, Alemania 2004.	Estudio de caso. Niña de 7 años con estreñimiento crónico y convulsiones.	Describir el tratamiento de una niña de 7 años con estreñimiento crónico y epilepsia desde la perspectiva de la euritmia terapéutica, de la enfermería y de la medicina.	Hospital Antroposófico
E2^13^	Dach C, Heine R, Heiligtag HR. Atención antroposófica a pacientes con cáncer, Alemania 2009.	Estudio de caso. Paciente de sexo femenino con diagnóstico de cáncer de mama ductal invasivo en el lado izquierdo, sometida a mastectomía conservadora y linfadenectomía.	Describir las terapias de enfermería en el tratamiento de pacientes con cáncer en todas las fases de la enfermedad.	Clínica Antroposófica
E3^14^	Stüdemann G. Compresas de borago para el linfedema. Observaciones desde la perspectiva de enfermería, Alemania 2011.	Investigación cualitativa mediante cuestionario. Diecisiete pacientes oncológicos con linfedema.	Identificar la percepción de bienestar de pacientes con linfedema tras el tratamiento con compresa de *Borago officinalis*.	Hospital Antroposófico
E4^15^	Ribeiro RM. El cuidado ampliado por la antroposofía: un estudio de caso sobre la práctica de la enfermería Antroposófica, Brasil 2013.	Estudio de caso con observación participante. Equipo de enfermería (1 enfermera y 1 técnica de enfermería).	Describir el ejercicio de la Enfermería Antroposófica a partir de su práctica y experiencia laboral en una clínica médica antroposófica; identificar los tratamientos adoptados en el cuidado de Enfermería Antroposófica; analizar los fundamentos del cuidado ejercido por la enfermería en el ser humano.	Clínica Antroposófica
E5^16^	Therkleson T. Tratamiento tópico de jengibre con compresas o parches para los síntomas de la osteoartritis, Nueva Zelanda 2014.	Estudio aleatorizado. Veinte adultos con osteoartritis crónica.	Evaluar y comparar la percepción de los participantes acerca de la intensidad del dolor, fatiga crónica, efecto global de la osteoartritis, movilidad y satisfacción con la salud; uso de medicación para el dolor y/o control del dolor; seguridad de la aplicación del adhesivo y de la compresa de jengibre.	Clínica Antroposófica y domicilio
E6^17^	Therkleson T. Terapia con jengibre para la osteoartritis: un caso típico, Nueva Zelanda 2014.	Estudio de caso. Paciente con osteoartritis crónica.	Describir el tratamiento para osteoartritis con terapia de jengibre, aplicado por enfermeros antroposóficos para un tipo específico de personalidad.	Clínica Antroposófica
E7^18^	Therkleson T. La búsqueda de un niño de 12 años de sí mismo: informe de un caso, Nueva Zelanda 2014.	Estudio de caso. Niño de 12 años con trastorno de estrés postraumático complejo (C-PTSD).	Describir los efectos del tratamiento de enfermería antroposófica en un niño con estrés postraumático complejo.	Clínica Antroposófica
E8^19^	Kusserow M. Aplicaciones externas en prácticas y clínicas antroposóficas, Alemania 2014.	Estudio de caso. Niño de 5 años con neumonía bilateral.	Describir el tratamiento antroposófico para un niño de 5 años con neumonía bilateral viral.	Unidad de Homeopatía de un Hospital Regional
E9^20^	Therkleson T, Stronach S. Síndrome del corazón roto: un caso típico, Nueva Zelanda 2015.	Estudio de caso. Mujer de 82 años con “Síndrome del Corazón Roto”, agotamiento general y ataques de ansiedad.	Describir el tratamiento para cardiomiopatía de Takotsubo “Síndrome del Corazón Roto”.	Centro Médico y domicilio
E10^21^	Ström M. Deslizamiento Rítmico según Wegman/Hauschka - un estudio de entrevistas sobre las experiencias de los pacientes con la terapia del toque en la atención ambulatoria, Suecia 2016.	Análisis de contenido. Cinco pacientes mayores de 18 años.	Aclarar cómo los pacientes que recibieron deslizamiento rítmico en atención ambulatoria vivenciaron el tratamiento y cómo los afectó.	Ambulatorio Antroposófico
E11^22^	Deckers B, Schoen-Angerer T, Voggenreiter B, Vagedes J. Aplicaciones externas de enfermería en el manejo de soporte del íleo postoperatorio prolongado: descripción de intervenciones y reporte de caso, Alemania 2016.	Reporte de caso. Paciente de sexo masculino de 61 años con íleo posoperatorio prolongado.	Describir el abordaje antroposófico de la enfermería para el tratamiento del íleo posoperatorio prolongado.	Hospital Antroposófico
E12^23^	Fujiwara-Pichler E, Beckmann U, Madeleyn R. Neumonía bacteriana adquirida en la comunidad en un niño tratado sin antibióticos en un hospital de medicina antroposófica: reporte de caso, Alemania 2020.	Estudio de caso. Niño caucásico de 4 ½ años con neumonía bacteriana adquirida en la comunidad (PAC), precedida por infección mixta de garganta viral y estreptocócica.	Describir el tratamiento de neumonía bacteriana adquirida en la comunidad en un niño tratado sin antibióticos en un hospital de medicina antroposófica.	Hospital Antroposófico

Los cuidados de enfermería ampliados por la antroposofía se caracterizan en la Figura 4, mediante la visualización del tipo, la duración y la frecuencia, los resultados y las recomendaciones descritos en los estudios.

**Figura 4:** Caracterización de las intervenciones de enfermería según tipo, duración, frecuencia, resultados y recomendaciones. Brasilia, DF, Brasil, 2024

Código del estudio	Dato clínico	Tipo, duración y frecuencia de los cuidados de enfermería	Resultados y recomendaciones
E1^12^	Niña de 7 años con estreñimiento crónico y convulsiones.	Deslizamiento abdominal con pomada *Oxalis folium* 10% en círculo alrededor del ombligo durante unos 5 minutos; baño de dispersión de aceite de lavanda al 10% con descanso de 10 minutos después del baño; deslizamiento con aceite de lavanda al 10% en piernas y pies con 10 minutos de descanso. Los baños de dispersión se realizaron en días alternos; el deslizamiento abdominal se aplicó todos los días antes del almuerzo; y el deslizamiento en piernas y pies todos los días durante el periodo de hospitalización.	Los cuidados de enfermería combinados con arteterapia, masaje rítmico, elementos de educación curativa, conversación orientada a la situación familiar y tratamiento médico permitieron que, tras tres semanas de hospitalización, el estreñimiento se curara completamente, con mejora de las convulsiones y armonización de la situación familiar.
E2^13^	Mujer con cáncer de mama sometida a mastectomía conservadora y linfadenectomía.	En la primera semana de hospitalización se realizó: deslizamiento rítmico en la espalda con aceite de *Solum ulioginosum*; compresa en el hígado con té de *Achillea millefolium* después del almuerzo; envoltura en el brazo izquierdo con aceite de *Aconitum*; compresa de árnica en ambas mamas para aliviar tensión, dolor y sensación de peso en las mamas. En la segunda semana de hospitalización: deslizamiento rítmico en la espalda y envoltura de queso Quark en región de mama y tórax izquierdo. Los gestos de enfermería antroposófica identificados en las dos primeras semanas fueron aliviar, envolver, confortar; en las semanas siguientes fueron crear espacio y aliviar.	La paciente presentó mejoría del dolor en brazo, mama y espalda, y mejoría de la sensación de tensión y peso en el brazo y la mama izquierda, sin necesidad de analgésicos y medicamentos para dormir en el momento del alta clínica. Se enseñó la realización de compresas y envolturas para su ejecución en domicilio para dolor en la región cervical. Se recomiendan las terapias externas antroposóficas como un complemento y una extensión de la atención oncológica.
E3^14^	Paciente oncológico con linfedema.	Aplicación de compresa con Esencia de *Borago officinalis L*. 20% durante 30 minutos en el miembro afectado, y tras la aplicación, pomada de *Angelica archangelica* en las extremidades congestionadas, una vez al día, durante 2 a 3 semanas o hasta la reducción del edema.	La aplicación de compresas y pomadas aportó alivio significativo de los síntomas derivados del linfedema, así como la percepción corporal de estar “más ligero”, “relajado”, “pierna más delgada” y “revigorizado”.
E4^15^	Actuación de enfermería en clínica Antroposófica.	Realización de baño intestinal con té compuesto de hierbas, bicarbonato de sodio, sal gruesa, sílice y fórmica; baño de dispersión de aceite; compresa en los órganos con soluciones y tés; baño de pies con tés, tinturas y sales; deslizamiento corporal; envoltura de todo el cuerpo con sábana calentada; administración parenteral de medicamentos. El tiempo de aplicación de las terapias varió según la terapéutica aplicada, en promedio de treinta a sesenta minutos. Tras la aplicación de la terapia, el paciente permanecía en un ambiente de reposo y silencio para que ocurriera la fase regenerativa. La frecuencia de las intervenciones era diversificada, dirigida por la prescripción médica.	Las terapias identificadas fueron el baño intestinal, compresas, deslizamiento corporal, baños de inmersión con aceite, envolturas, baño de pies y administración de la terapia con *Viscum album* en pacientes oncológicos. El cuidado se fundamentó en los doce sentidos, en los siete procesos vitales, en los doce gestos del cuidado, en la trimembración y en la cuatrimembración. Se destaca al paciente como foco del cuidado y que el proceso de curación implica la existencia de fuerzas sutiles que pueden ser estimuladas por el cuidado de enfermería.
E5^16^	Pacientes adultos con osteoartritis crónica.	Aplicación de adhesivo de material orgánico seco molido del rizoma de *Zingiber officinale*, o compresa de jengibre en la región medio-lumbar durante 30 minutos, seguida de un descanso de 20 minutos. Aplicación tópica una vez al día de adhesivo o compresa de jengibre durante 7 días consecutivos; autoaplicación tópica de adhesivo de jengibre en domicilio durante 24 semanas.	Tras 1 semana de tratamiento tópico con jengibre hubo descenso en las puntuaciones de dolor, fatiga, efecto global y estado funcional, mientras que la satisfacción con la salud mejoró de 80% de insatisfacción a 70% satisfechos. Las puntuaciones en los cinco dominios se redujeron progresivamente a lo largo de las 24 semanas de autotratamiento, así como la reducción del uso de analgésicos, llegando a la no utilización de medicación para el dolor por la mayoría de los participantes al final del periodo de estudio. Se recomienda el tratamiento tópico con jengibre, pues su aplicación es sencilla y económica, debiendo considerarse en el cuidado de personas mayores con osteoartritis crónica.
E6^17^	Paciente con osteoartritis.	Aplicación de compresa de jengibre en la región medio-lumbar (renal) durante 30 minutos, seguida de un descanso de 15 minutos, por 7 días consecutivos, y aplicaciones continuas dos o tres veces por semana para mantener los niveles de dolor.	La compresa de jengibre aportó alivio inmediato y progresivo de los síntomas de osteoartritis a lo largo de 24 semanas, sin efectos negativos referidos por el paciente. Se recomienda el uso de la compresa de jengibre en el cuidado de enfermería a pacientes con osteoartritis.
E7^18^	Niño de 12 años con trastorno de estrés postraumático complejo.	Terapia combinada de baño de pies con limón, deslizamiento rítmico corporal con aceite *Solum ulioginosum*, deslizamiento cardíaco con pomada *Aurum*-Lavanda-Rosa; deslizamiento en pentagrama con pomada *Aurum*-Lavanda-Rosa. Las terapias se aplicaron una vez por semana durante 5 semanas, en sesiones de 60 minutos, con 15 minutos de descanso entre las terapias.	El baño de pies, el masaje cardíaco y los deslizamientos rítmicos, indicados para los síntomas de trastorno de estrés postraumático complejo, solo surtieron efecto tras la inclusión del deslizamiento en pentagrama. Se recomienda que el deslizamiento en pentagrama sea considerado en el manejo de trauma, estrés, agotamiento y desconexión en niños de 12 años.
E8^19^	Niño de 5 años con neumonía bilateral.	Aplicación de gel de *Cochlearia armoracia* en los senos nasales; compresa de *Achillea millefolium*, con *Petacites officinalis* en región torácica, durante 45 minutos, una vez al día, por 14 días. Compresa de limón en momentos de fiebre.	Las compresas aportaron alivio de los síntomas de tos y esfuerzo para respirar, relajación para dormir, recuperación del apetito, mejoría del estado de ánimo - poniéndose más contento - y autorregulación de la temperatura.
E9^20^	Mujer de 82 años con “Síndrome del Corazón Roto”.	Sesiones de baño de pies con lavanda, deslizamiento rítmico usando aceite *Solum ulioginosum* y cataplasma caliente de pomada *Oxalis folium*, una sesión por semana durante 4 semanas. En domicilio, durante el tratamiento, la paciente recibió una “almohada cardíaca” impregnada con pomada de *Aurum* Lavanda-Rosa, para ser aplicada sobre el seno izquierdo por la noche, uso diario durante 4 semanas.	El tratamiento con baño de pies y deslizamiento rítmico no interfirió en la respuesta a los medicamentos en uso y permitió que la persona mayor traumatizada pudiera superar el miedo y encontrar un coraje renovado para responder a su mundo y al de los demás. Se recomienda el uso de baño de pies y deslizamiento rítmico cuando hay opciones limitadas para gestionar traumas y estrés.
E10^21^	Pacientes que recibieron deslizamiento rítmico.	Sesiones de deslizamiento rítmico en entorno ambulatorio, aplicadas 1 o 2 veces por semana, durante 4 a 8 semanas.	El deslizamiento rítmico aportó mejoría de la función pulmonar (tos, asma y enfermedad pulmonar obstructiva crónica), función intestinal, dolor de cabeza, dolor general en condiciones de dolor prolongado y sueño. Experimentar el cuidado ofrecido fue descrito como sentir estabilidad, seguridad, sentimiento de conexión y relajación. La experiencia de ser confirmado fue descrita como recuperar los límites, ser visto, sentirse, sentir significado y participante de un contexto. La vitalidad se expresó como experiencias de sentirse más fuerte, con esperanza y con más energía. Experiencias de salud style="padding:10px; border:1px solid black" a pesar del sufrimiento y enfermedad grave fueron descritas como sentirse bien y tranquilo en una situación de vida caótica.
E11^22^	Paciente con íleo posoperatorio prolongado.	Aplicación de compresa con tintura de *Oxalis folium* 20% en la región abdominal durante 20 minutos, seguida de 30 minutos de descanso; realización de masaje con aceite de melisa en el abdomen, en sentido horario, durante unos 5 minutos; aplicación de compresa con pomada *Thuja occidentalis* 10% y *Argentum Metallicum* 0,4%, en cantidades iguales, en el abdomen durante 30 minutos, seguida de 30 minutos de descanso. Durante 10 días, 1 vez por la mañana, se aplicó compresa de *Oxalis folium* seguida de masaje con aceite de melisa; y 1 vez por la noche, compresa de pomada *Thuja*/ *Argentum*.	El uso de compresas y masajes abdominales mejoró la sensación de bienestar y redujo el dolor, siendo posible gestionar el íleo posoperatorio prolongado sin fármacos procinéticos. Se recomienda que compresas y masaje ligero en región abdominal se utilicen como herramientas adicionales en el manejo del íleo posoperatorio prolongado.
E12^23^	Niño de 4 ½ años con neumonía bacteriana adquirida en la comunidad.	Aplicación de compresa tibia de queso tipo Quark en la pared torácica derecha, durante 20 minutos en el 1º día de tratamiento; aplicación de compresa de aceite de lavanda tibia en la pared torácica anterior, una vez al día durante 6 días; aplicación de bolsa de agua caliente para hipotermia tras medicación rectal para la fiebre; del 2º al 6º día de tratamiento recibió compresa de mostaza molida en la pared torácica derecha, durante 2 a 3 minutos.	La neumonía fue tratada de forma eficaz y segura, sin el uso de antibióticos, mediante pediatras preparados para reevaluar al niño con frecuencia, equipo de enfermería familiarizado con este tratamiento y apoyo de los padres. Se recomienda que las terapias se administren en instalaciones apropiadas y especializadas para este tipo de tratamiento, incluyendo personal altamente capacitado para reducir el riesgo de complicaciones, incluida la muerte.

## Discusión

Entre los estudios analizados en esta revisión, en lo que se refiere al idioma, seis fueron redactados en lengua inglesa, cuatro en alemana, uno en portugués y uno en sueco. En relación con el tipo de publicación, diez corresponden a artículos científicos, uno a disertación de maestría y uno a tesis doctoral. En cuanto al origen geográfico, cuatro estudios provienen de Nueva Zelanda, seis de Alemania, uno de Brasil y uno de Suecia^12^ ^-^ ^23^.

Esta revisión permitió mapear las características del cuidado ampliado por la antroposofía, evidenciando que la EA utiliza terapias externas, expresadas en las prácticas de compresas, cataplasmas, envolturas, baños, baño de pies, lavados intestinales, masaje con miel, deslizamientos rítmicos, pentagrama, administración parenteral e inhalatoria de medicamentos, usadas para quejas físicas (dolor, tos, asma, estreñimiento, dificultad para respirar, convulsiones, tensión muscular, fatiga, falta de apetito, dificultad de autorregulación de la temperatura, neumonía) y anímicas (miedo, sufrimiento psíquico, insatisfacción con la salud, estrés, agotamiento mental, estado de ánimo deprimido). Estas terapias combinan atención interpersonal, toque, movimientos rítmicos, mantenimiento del calor, elementos fitoterápicos (aceites, esencias, tinturas, pomadas) en intervenciones aplicadas diariamente o 1-2 veces por semana, en sesiones de 30-60 minutos, con duración dependiendo de la sustancia aplicada, que son seguidas por un periodo de reposo terapéutico, y administradas en ciclos de tratamiento de acuerdo con la condición clínica del paciente^12^ ^-^ ^23^.

En los estudios analizados, las terapias externas se asociaron a la terapia medicamentosa antroposófica al inicio del tratamiento, con el objetivo de estimular la autorregulación del calor corporal y la movilización de las fuerzas etéricas, siendo el vehículo para el acceso de los medicamentos a los otros cuerpos, anímico y Yo, contribuyendo a que la medicación oral surta efecto más rápidamente y movilice fuerzas que auxilien en el proceso de curación^12^ ^,^ ^14^ ^,^ ^16^ ^,^ ^18^ ^-^ ^20^ ^,^ ^22^ ^-^ ^23^. En un primer momento las terapias externas son coadyuvantes en el proceso terapéutico, pero, con el paso de las sesiones, se tornan la terapia curativa principal^13^ ^,^ ^16^ ^-^ ^17^. Las terapias externas son señaladas en la literatura como facilitadoras de la autorregulación autonómica y de procesos salutogénicos (que promueven y potencian el desarrollo de un buen estado subjetivo de salud), influyendo físicamente en la distribución del calor en el organismo, integrando las funciones de cuerpo, alma y espíritu, por medio de la regulación de procesos vitales, como respiración, calentamiento, nutrición, excreción, mantenimiento, crecimiento y reproducción, así como de la armonización de las cuestiones emocionales y de procesos de significación de la vida y de la salud^2^ ^,^ ^24^.

Los estudios señalan al individuo como foco del cuidado en la EA, mediante el reconocimiento de su naturaleza cuádruple^12^ ^-^ ^23^. La literatura explica que, al observar cómo los aspectos de esta naturaleza cuádruple interactúan entre sí y cómo se expresan, se alcanza un nuevo nivel de comprensión y ocurre un proceso de evaluación empírica de los cuerpos más sutiles. Por ejemplo, la funcionalidad del cuerpo vital, que mantiene todas las partes en una relación viva, puede observarse en el equilibrio de fluidos, en los ciclos de sueño/vigilia y en la calidad del cabello y de las uñas. La salud del cuerpo emocional puede evaluarse por el equilibrio emocional, las experiencias de dolor y las fuertes reacciones a las circunstancias o a las personas con simpatía o antipatía. Por último, la presencia del “Yo” como activo en el individuo puede observarse a través del sentido de coherencia que se tiene en la vida, de la conexión con la espiritualidad y/o con el mundo, de la capacidad de volverse observador tanto de los otros como de sí mismo y de la capacidad de encontrar significado en la vida o en la enfermedad. Esto proporciona al enfermero antroposófico una comprensión profunda de los otros y de sí mismo y, naturalmente, pasa a orientar el cuidado prestado^4^ ^,^ ^24^.

Los estudios destacaron que el cuidado de EA se ofrece a pacientes en los diferentes ciclos de vida, con condiciones clínicas diversas, agudas o crónicas, con intervenciones que producen impactos inmediatos y a corto plazo en la salud, en entornos de atención primaria, atención ambulatoria y hospitalaria, y que pueden integrarse con prácticas de salud de la medicina convencional^12^ ^-^ ^23^. También se resaltó la toma de decisiones compartida y el énfasis en la autogestión de síntomas relacionados con la enfermedad, con resultados a medio y largo plazo, viabilizada por la posibilidad de que algunas terapias externas sean enseñadas para su autoaplicación en domicilio^13^ ^,^ ^16^ ^-^ ^17^ ^,^ ^20^, principalmente a pacientes que enfrentan síntomas crónicos, debido a la naturaleza simple de ejecución y al bajo costo de sus elementos terapéuticos.

En otro ámbito los cuidados fueron evidenciados, como en las enfermedades crónicas en niños. Se consideró que las enfermedades crónicas derivan del modo de vida actual y de experiencias sensoriales y mentales en la infancia, y pueden tratarse con la terapia antroposófica, principalmente cuando otras terapias se han intentado sin éxito. El objetivo es sustituir influencias que causan enfermedades por otras que preservan la salud. La integración mutua de terapias de enfermería, terapia artística, deslizamiento rítmico, educación curativa, debates orientados con la familia y terapia farmacológica colaboran para crear un equilibrio en la percepción empática de la carga familiar individual y una identificación clara de las condiciones y consecuencias de las influencias de la vida cotidiana familiar en la salud de los niños. En este contexto, se reconoce que en ocasiones el tratamiento en entorno ambulatorio no surte el efecto deseado, siendo necesario seguimiento en entorno hospitalario, para que los aspectos físicos y anímicos puedan ser comprendidos y acompañados con un plan terapéutico más asertivo para el niño y el contexto familiar^12^.

El cuidado de EA en el área de oncología fue resaltado mediante terapias externas como compresas, envolturas, deslizamientos rítmicos según Wegman y Hauschka, medicamentos antroposóficos, orientaciones de cuidados de salud para el domicilio y actitud interior del profesional de enfermería, que constituyen un complemento a la atención oncológica. La terapéutica en el contexto oncológico se basó en la promoción de calor interno, para que se establezca la integración entre los cuatro cuerpos del ser humano y se estimule el principio curativo. Se destaca que las terapias externas ofrecidas provienen de la decisión del profesional de enfermería, sustentado en los doce gestos de la enfermería antroposófica, que ofrecen un modelo de evaluación, orientación y ejecución del plan terapéutico de enfermería^13^ ^-^ ^14^.

Los aspectos curativos del cuidado de EA y la participación del paciente son señalados en la literatura como actividades intangibles que contribuyen a resultados y experiencias positivas del paciente, y necesitan ser valoradas en los contextos de salud con igual importancia que los procesos terapéuticos convencionales de curación^4^.

Los estudios destacaron que el nivel de conciencia que los profesionales de enfermería comparten en sus intervenciones eleva el trabajo de enfermería más allá de modelos mecánicos, a un nivel terapéutico, que incluye las cualidades internas intangibles que los profesionales aportan a la experiencia del cuidado, es decir, la ampliación de la conciencia no sobre lo que el profesional de enfermería hace por el paciente, sino cómo se hace. Este proceso posibilita al profesional comprender de qué forma el cuidado ofrecido afecta a los cuatro cuerpos del paciente, además de influir en su estado de ánimo interno y en el estado de ser del propio profesional. Cada encuentro es visto como único, portador del potencial de transformación para todos los involucrados en el acto de cuidado. La intención consciente puede ejemplificarse por actitudes expresadas en 12 gestos de la enfermería, que fortalecen al profesional y apoyan al paciente^13^ ^,^ ^15^ ^,^ ^19^ ^,^ ^21^. Los doce gestos de Enfermería Antroposófica se clasifican en dos grupos: gestos envolventes y gestos activadores. Los gestos envolventes buscan satisfacer una necesidad que la persona, en determinada condición, no es capaz de suplir de manera autónoma. En este abordaje, el profesional actúa de forma interventiva, en nombre de la seguridad o el confort del paciente. Por su parte, los gestos activadores tienen como objetivo estimular a los pacientes a tomar conciencia y actuar en su propio nombre, favoreciendo el rescate de su capacidad interna de reacción y autorregulación^4^.

Ante los hallazgos, la limitación de este estudio es su carácter de alcance, explorando publicaciones que describieran los cuidados de enfermería ampliados por la antroposofía, lo que limitó la expresividad de la revisión, pues algunas investigaciones enfocaban las terapias externas, pero con diseños metodológicos poco claros que no permitían identificar si la terapia referida era utilizada en la práctica de enfermería. Se recomienda que estudios futuros se centren en un abordaje metodológico objetivo y claro, y amplíen el escenario de actuación de la EA al ámbito de gestión, de enseñanza y de especialidades en el área de salud, referenciadas en la literatura, pero que no fueron evidenciadas, como por ejemplo la Enfermería Obstétrica Antroposófica^1^.

Los resultados provenientes de esta revisión contribuyen al avance del conocimiento científico en el área de las Prácticas Integrativas y Complementarias, especialmente para la enfermería antroposófica. Esta área exige el perfeccionamiento de habilidades y conocimientos especializados, para que los profesionales puedan incorporar nuevas tecnologías y prácticas, además de competencias interpersonales esenciales para lidiar con las diversas situaciones que la profesión presenta. Las evidencias recogidas ayudan a llenar la brecha de conocimiento sobre el quehacer de la Enfermería Antroposófica y destacan la necesidad de futuras investigaciones que consoliden este conocimiento y amplíen las formas de cuidado de enfermería en diferentes contextos de salud.

## Conclusión

El mapeo de las características del cuidado de enfermería ampliado por la Antroposofía en los servicios de salud evidenció a la Enfermería Antroposófica como especialidad en el área de enfermería, con un quehacer basado en conocimiento propio proveniente de la Medicina Antroposófica, que posibilita la integración con prácticas de salud de la medicina convencional y la actuación en equipo multiprofesional. Presenta al individuo como foco del cuidado, en diferentes contextos de salud, ya sea hospitalario, de clínicas de salud o en domicilio, con condiciones clínicas diversas, agudas o crónicas.

Los cuidados de enfermería requieren del profesional conocimientos y habilidades específicas sobre las prácticas terapéuticas antroposóficas, para ofrecer una atención eficaz y segura en los diferentes contextos de salud y de vida. Estas prácticas tienen características de seguimiento a corto, medio y largo plazo, siendo que algunas pueden enseñarse al paciente para su autoaplicación en domicilio, como forma complementaria al plan terapéutico establecido. El destaque recae sobre la corresponsabilidad en el proceso terapéutico y en la postura del profesional de enfermería percibida por los pacientes como factor facilitador del proceso de curación. La Enfermería Antroposófica amplía la mirada sobre la promoción de la salud y las necesidades individuales de los pacientes que, en ocasiones, están más allá de los límites de la medicina convencional.

## Declaración de Disponibilidad de Datos:

Todos los datos generados o analizados durante este estudio están incluidos en este artículo publicado.
